# BrainXcan identifies brain features associated with behavioral and psychiatric traits using large-scale genetic and imaging data

**DOI:** 10.1016/j.dcn.2025.101542

**Published:** 2025-03-13

**Authors:** Yanyu Liang, Festus Nyasimi, Owen Melia, Timothy J. Carroll, Thomas Brettin, Andrew Brown, Hae Kyung Im

**Affiliations:** a Section of Genetic Medicine, University of Chicago, Chicago, IL, United States; b Department of Computer Science, University of Chicago, Chicago, IL, United States; c Department of Radiology, University of Chicago, Chicago, IL, United States; d Computing Environment and Life Sciences Directorate, Argonne National Laboratory, Lemont, IL, United States; e Consortium for Advanced Science and Engineering, University of Chicago, Chicago, IL, United States; f Department of Population Health and Genomics, University of Dundee, Dundee, United Kingdom

**Keywords:** Image derived phenotypes, Association study, Complex phenotype genetics, Mendelian Randomization, Genetic architecture, Genetic prediction

## Abstract

Advances in brain MRI have enabled many discoveries in neuroscience. Case-control comparisons of brain MRI features have highlighted potential causes of psychiatric and behavioral disorders. However, due to the cost and difficulty of collecting MRI data, most studies have small sample sizes, limiting their reliability. Furthermore, reverse causality complicates interpretation because many observed brain differences are the result rather than the cause of the disease. Here we propose a method (BrainXcan) that leverages the power of large-scale genome-wide association studies (GWAS) and reference brain MRI data to discover new mechanisms of disease etiology and validate existing ones. BrainXcan tests the association with genetic predictors of brain MRI-derived features and complex traits to pinpoint relevant brain-wide and region-specific features. Requiring only genetic data, BrainXcan allows us to test a host of hypotheses on mental illness, across many MRI modalities, using public data resources. For example, our method shows that reduced axonal density across the brain is associated with schizophrenia risk, consistent with the disconnectivity hypothesis. We also find that the hippocampus volume is associated with schizophrenia risk, highlighting the potential of our approach. Taken together, our results show the promise of BrainXcan to provide insights into the biology of GWAS traits.

## 1 Introduction

Advances in MRI technology have enabled the measurement of brain structure, connectivity, and other features in a noninvasive manner. Many studies have successfully examined differences in brain features between diseased and healthy individuals to learn about the pathophysiology of brain-related diseases. However, the difficulty of recruiting patients and controls and the cost of the MRI scans have limited the sample sizes of these studies, greatly reducing their reproducibility (Marek et al., 2020).

On the other hand, genome-wide association studies (GWAS) use a very large number of participants (in some cases more than a million) to discover genetic mutations that affect brain-related diseases. For example, the Psychiatric Genomics Consortium (PGC) has performed GWAS of 11 psychiatric disorders, including attention-deficit/hyperactivity disorder, Alzheimers disease, autism, bipolar disorder, and schizophrenia, to elucidate their genetic basis. However, since these studies do not typically include brain imaging data, comparison of MRI images between cases and controls is not possible.

To bring together the advantages of the sophisticated and deep phenotypic data available in imaging studies and the very large sample sizes of GWAS studies, we turned to integrative approaches, such as transcriptome-wide association studies (TWAS). TWAS leverage GWAS and eQTL studies to genetically predict gene expression levels, test their correlation with complex traits and diseases, and ultimately, identify potentially causal genes (Gamazon et al., 2015, Gusev et al., 2016). Theoretically, this strategy could be applied to predict brain-imaging phenotypes instead of gene expression data. However, the genetic variants regulating the expression of a given gene often have large effect sizes and are concentrated near the genes transcription start site (Wheeler et al., 2016). These properties limit the number of large-effect causal variants and make them easy to detect, simplifying prediction. In contrast, genetic predictors of brain features may be distributed throughout the genome, and their individual effect sizes are likely to be quite small, thus making them more difficult to utilize with current prediction methods.

Here we propose BrainXcan, an extension of PrediXcan, that uses GWAS data (individual-level or summary statistics) to predict and compare image-derived brain features in cases and controls (Gamazon et al., 2015). Rather than relying on a simple polygenic risk score to predict brain features (Knutson et al., 2020) or genetic correlations (Zhao et al., 2021), BrainXcan performs a comprehensive analysis of the genetic architecture to optimize prediction, which enhances both power and interpretability. Here, we apply BrainXcan to several complex traits using both large-scale GWAS summary statistics and UK Biobank’s individual-level data with close to half a million individuals. We demonstrate that BrainXcan can address the computational challenges and statistical issues stemming from the high polygenicity and genome-wide scale. Further, we show that BrainXcan can be used to corroborate existing hypotheses and generate new hypotheses about the role of brain function and structure on psychiatric and neurological disorders.

## 2 Results

### 2.1 Overview of the BrainXcan framework

**Fig. 1:**
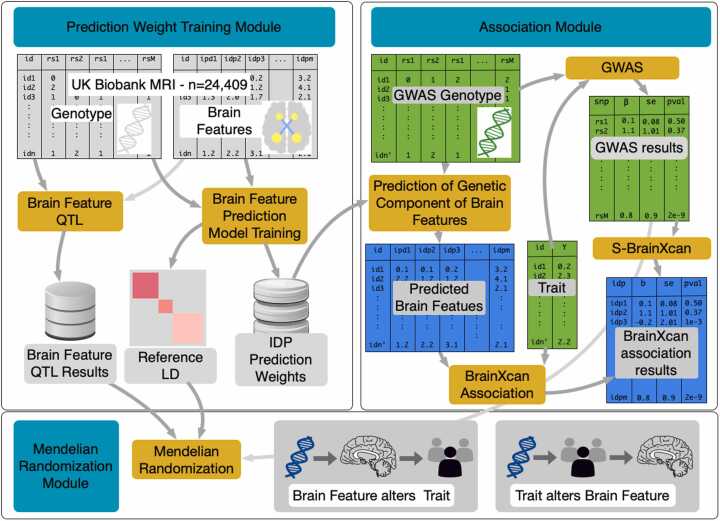
Overview of BrainXcan. The workflow is divided into three modules: prediction weight training (top left), association (top right), and Mendelian randomization (bottom). The prediction weight training model has been completed and results are provided as part of the software package to predict brain features on user-provided genotype data. The association module uses a TWAS-like method to find associations between predicted brain features and user-provided phenotype data. The Mendelian randomization model seeks to elucidate putative causal flow of brain featuretrait associations.

The BrainXcan framework is organized into three modules, prediction weight training, association, and Mendelian randomization, as outlined in Fig. 1. The prediction weight training module trains linear genetic predictors of brain features, tests for association between brain features and genotype (image-derived phenotype prediction weights), and calculates the sample covariance of the genotypes (reference LD). The training does not need to be repeated by end users; these outcomes are saved for use in subsequent modules and shared publicly in predictdb.org with versioned and permanent records in zenodo.org (Liang et al. 2021).

The association module uses the brain feature prediction weights and reference LD information produced by the prediction weight training module in conjunction with user-provided genotype and trait data (individual- or summary-level) to compute the BrainXcan association results table, which will provide estimated coefficients of the regression between the trait and the genetically predicted brain features, their standard error, and p-values. Significant associations pinpoint candidate causal relationships between brain features and the trait, which may be due to direct effects or shared genetic factors that influence both traits. As will be explained in the brain feature processing section, we used features that represent both region-specific and brain-wide measurements.

Finally, the Mendelian randomization module performs a number of Mendelian randomizations based on multiple instruments to examine the direction of the putative causal flow, i.e., whether alterations in brain features affect the complex trait or whether the trait (e.g., disease status) alters brain features. We discuss each module in depth below, including technical challenges we addressed while extending existing association methods to image-derived features.

### 2.2 Prediction weight training module

#### 2.2.1 Selection and preprocessing of brain MRI derived features

We downloaded uniformly preprocessed brain image-derived phenotypes (IDPs) obtained via structural and diffusion MRI from the UK Biobank. We selected 159 features derived from structural images, representing total and gray matter volumes from different regions of the brain, and 300 diffusion MRI-derived features, representing neurite density, dispersion, and connectivity features. All of these diffusion MRI features were included in the genetic architecture analysis and in the BrainXcan software; however, for the ease of description, in this paper we focused on the 192 diffusion features mapped to the Johns Hopkins University’s 48-region atlas tracts (referred to here as “main diffusion features”) (Wakana et al., 2004). After excluding related individuals and those of non-European ancestry, brain features from 24,409 individuals remained (Table S1). We adjusted each feature for covariates including the first 10 genetic principal components (PCs), age at recruitment, sex, and four technical covariates indicating the location of the head in the scanner. See details in Methods and the list of brain features in Table S2.

We processed the structural and diffusion MRI modalities separately. We categorized structural features into five subtypes: cortical gray matter volume, subcortical gray matter volume, subcortical total volume, cerebellum gray matter, and brain-stem. We also considered total gray matter volume and total gray and white matter volume as additional structural features. We categorized diffusion MRI measures into four subtypes: fractional anisotropy (FA, a measure of diffusion along the white matter tracts), intracellular volume fraction (ICVF, an estimate of neurite/axonal density), isotropic volume fraction (ISOVF, an index of the relative extra-cellular water diffusion), and orientation diffusion index (OD, a measure of neurite dispersion)(Zhang et al., 2012).

For each subtype, we performed a principal components analysis. The first PC was a weighted average of features in the subtype, suggesting that principal components could be used as proxies for the brain-wide features (Fig. S1). For the structural MRIs, the first principal component explained 16 % (cortical gray matter), 34 % (subcortical gray matter), 37 % (subcortical total volume), and 45 % (cerebellum gray matter) of the total variability. For diffusion MRI subtypes, the first principal component explained 39 % (FA), 53 % (ICVF), 26 % (ISOVF), and 20 % (OD) of the total variance. As expected, after adjusting for the first principal component within each subtype, the correlation structure of the residual features was substantially reduced (Figs. S2 and S3).

#### 2.2.2 Generative models of brain features and complex traits

Since an important attribute of any method is the interpretability of the results, we sought to define brain features with the goal of facilitating interpretation. We prioritized the ability to tease out brain-wide effects from region-specific effects. i.e., determining whether a detected association with the trait was due to a feature that is common across the whole brain or specific to a region.

**Fig. 2:**
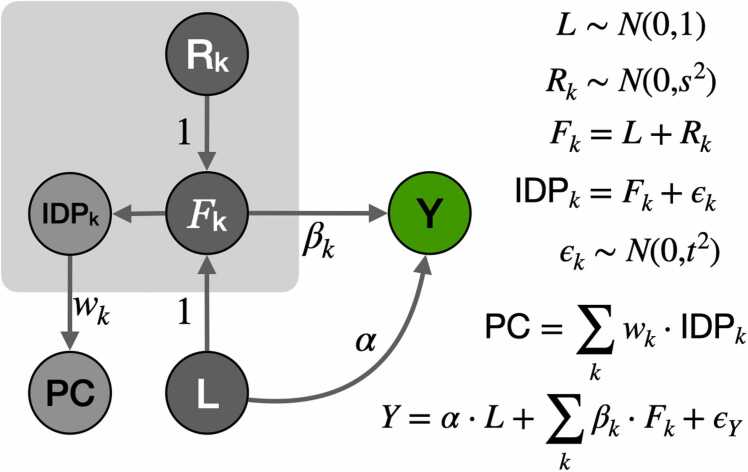
Generative model of brain features and complex traits. Brain features, $F_{k}$, are modeled as a sum of brain-wide component L and a region-specific effect $R_{k}$. The observed brain features (IDPs) are considered to be noisy measurements of true features, $F_{k}$. PCs of IDPs are weighted averages of IDPs. The complex trait Y is modeled as the sum of brain-wide effect (α) and region-specific effects ($\beta_{k}$) and an error term ($\epsilon_{Y}$).

To distinguish between brain-wide and region-specific effects, we postulated the generative model shown in Fig. 2. The brain feature in each region ($F_{k}$) is modeled as the sum of two independent latent components: a brain-wide component (L) and a region-specific component ($R_{k}$). The observed value, IDP, is modeled as a noisy version of the region’s feature (${\mathrm{IDP}}_{k}=F_{k}+\epsilon_{k}=L+R_{k}+\epsilon_{k}$). The parameter $s^{2}$ determines the scale of the region-specific component (modeled as a normal random variable with variance $s^{2}$) and $t^{2}$ is the variance of the noise term $\epsilon_{k}$. Since we ultimately want to assess the significance of the association between predicted brain features and traits, multiplying the traits by a common scaling factor will not change the results. Accordingly, we chose the distribution of the latent variable L to have variance equal to one. PC is a weighted average of each region’s observed features, which we interpret as the brain-wide component of the IDPs.

Other approaches have incorporated additional PCs of raw brain features into their studies (Zhao et al., 2021). While we acknowledge that our decision to use only the first principal component was somewhat arbitrary, it simplifies the interpretability of our results and makes our method more user-friendly.

#### 2.2.3 Genetic architecture of brain features

To determine whether brain features could be predicted using genetic data alone and to better inform optimal prediction approaches, we proceeded to investigate their genetic architecture. We calculated the heritability (roughly the magnitude of the genetic component) and degree of polygenicity (a measure of the number of independently associated variants) of brain features (Table 1 and Table S3).

**Table 1:** A summary of the genetic architecture of prediction performance of brain IDPs. This table summarizes the genetic architecture estimates, heritability, and polygenicity (in terms of $M_{e}$ (O’Connor et al., 2019)), and the prediction performance of ridge models (in terms of $R^{2}$) of brain IDPs organized by IDP subtypes (defined in Table S2). **Modality** and **subtype** show the modality of subtype name (see definitions in Table S2). **#IDPs** shows the number of brain IDPs falling in the subtype. **h2**, **Me**, and **R2** show the average heritability, polygenicity, and prediction performance among the IDPs (excluding principal component) within the subtype and the corresponding standard deviation is shown inside the parentheses. **h2(PC)**, **Me(PC)**, and **R2(PC)** show the heritability, polygenicity, and prediction performance of the principal component of the subtype. Prediction performance is measured as the squared correlation between observed and predicted IDPs.

**modality**	**subtype**	**# IDPs**	**h2(PC)**	**h2**	**Me(PC)**	**Me**	**R2(PC)**	**R2**
T1	Gray-Cerebellum	28	0.38	0.28 (0.06)	6362	4789 (2553)	0.050	0.028 (0.009)
T1	Gray-Cortical	96	0.30	0.18 (0.07)	6896	10230 (14278)	0.030	0.012 (0.009)
T1	Gray-Subcortical	14	0.34	0.25 (0.06)	4922	5502 (2478)	0.039	0.023 (0.009)
T1	Subcortical	14	0.33	0.22 (0.09)	4829	11420 (15757)	0.039	0.019 (0.013)
T1	Total	5	NA	0.32 (0.02)	NA	6893 (1863)	NA	0.035 (0.006)
dMRI	FA	48	0.35	0.25 (0.05)	2784	8158 (4517)	0.042	0.023 (0.008)
dMRI	ICVF	48	0.43	0.26 (0.06)	559	6806 (6231)	0.060	0.024 (0.009)
dMRI	ISOVF	48	0.21	0.13 (0.06)	2002	8712 (15333)	0.017	0.008 (0.007)
dMRI	OD	48	0.34	0.26 (0.05)	3360	9036 (6116)	0.041	0.024 (0.009)
dMRI	w-FA	27	0.37	0.19 (0.05)	1803	4240 (25396)	0.047	0.014 (0.007)
dMRI	w-ICVF	27	0.43	0.24 (0.04)	500	8092 (6901)	0.061	0.021 (0.007)
dMRI	w-ISOVF	27	0.27	0.12 (0.04)	1513	1062 (33166)	0.025	0.006 (0.004)
dMRI	w-OD	27	0.33	0.19 (0.06)	3584	18715 (28648)	0.039	0.014 (0.009)

**Fig. 3:**
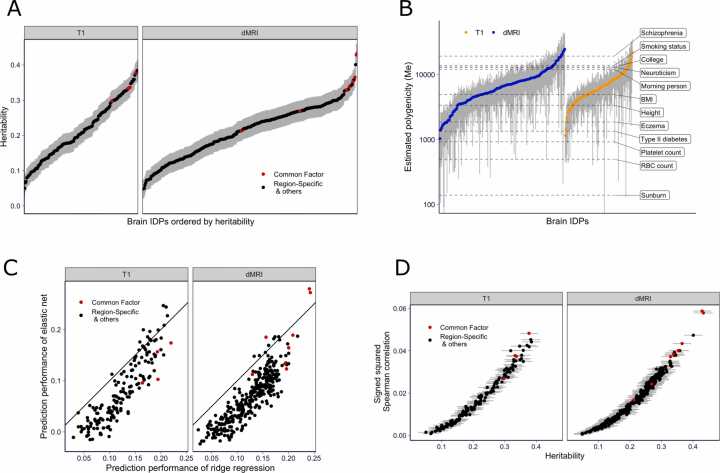
Genetic architecture and prediction performance of brain features. A) Heritability of brain features. Heritability and 95 % confidence intervals are shown for structural (T1) and diffusion (dMRI) features. Red denotes common factors (PCs of each subtype) and black denotes the residual features in the subtype (after regressing out PCs). B) Polygenicity of brain features. The effective number of independently associated SNPs ($M_{e}$) estimated using stratified LD fourth moments regression for 331 brain features are shown on the y-axis (IDPs with $M_{e}\leq 0$ or $M_{e}$ not significantly different from 0 are excluded). Gray bars indicate 95 % confidence intervals. Horizontal dashed lines indicate the estimated $M_{e}$ of 12 complex traits for reference. C) Prediction performance of brain features. This panel compares the performance of ridge regression vs. elastic net predictors measured by the Spearman correlation between the predicted and observed values in a five-fold cross-validated scheme. The performance of the ridge predictor is shown on the x-axis and the performance of the elastic net predictor is shown on the y-axis. The black solid line represents the identity line (y=x). The brain-wide features (IDP PCs) are shown in red, and the remaining brain features are shown in black. D) Prediction performance vs. heritability. The signed squared Spearman correlations between observed and predicted IDPs of the ridge regression are shown on the y-axis and the estimated heritability are shown on the x-axis. The signed squared correlation is defined as $\mathrm{sign}(x)\cdot x^{2}$ for correlation x to preserve the sign of the correlation while taking the square. The error bar indicates the 95 % confidence interval of the estimated heritability. The brain-wide features are in red, and the rest of the brain features are in black.

To calculate the heritability of brain features, we used a standard mixed effects modeling approach (Yang et al., 2010) (Methods). Heritability estimates ranged from 5 % to 43 % with all the 95 % confidence intervals above zero as shown in Fig. 3A. We note that the heritability of the principal components within each category (shown in red dots in Fig. 3A) is generally higher than the heritability of region-specific features (shown in black dots in Fig. 3A), providing support for our use of the principal components as proxies for brain features.

To quantify the degree of polygenicity of brain features, we estimated the effective number of independently associated SNPs ($M_{e}$) using stratified LD fourth moments regression (O’Connor et al., 2019) (Methods and Fig. S4). Among 471 IDPs (457 region-specific, and 14 brain-wide), 331 IDPs yielded a significant (p<0.05) estimate of $M_{e}$, with values ranging from 1035 to 24,675 and a median of 6251, which was higher than the estimates for canonical examples of polygenic traits such as height or BMI. The estimates are shown in Fig. 3B with common human traits added for reference.

These results indicate that the genetic prediction of brain features is feasible and suggest that predictors that include many genetic factors are likely to outperform sparser models with only a few genetic variants. Our findings are consistent with previous studies examining the heritability and polygenicity of brain features among similar cohorts (Elliott et al., 2018; Zhao et al., 2019; Biton et al., 2020; Smith et al., 2021; Matoba et al., 2022).

#### 2.2.4 Ridge regression and elastic net prediction training

To maximize the applicability of our method, we designed BrainXcan to use only linear models. Linear predictors allow BrainXcan to be applied using only GWAS summary statistics—even when the individual-level genotype and trait data are not available, as shown in Barbeira et al. (2018). This is because feature-level association can be closely approximated as linear combination of SNP-level associations when predictors are linear. To keep the computation manageable, we restricted the training to a common set of SNPs in HapMap 3 (minor allele frequency, or MAF > 0.01 in European samples and MAF > 0.05 in UKB IDP cohort), a subset of SNPs that tends to be imputed with high quality across many existing GWAS. To avoid issues with strand misspecification, we excluded the ambiguous SNPs (e.g. A/T, C/G). These restrictions left us with a total of 1.07 million SNPs (Methods) for subsequent analyses.

We then used these data to train two sets of predictors, one with a ridge and the other one with an elastic net penalty. Ridge regression uses $l_{2}$ penalty and yields highly polygenic predictors (all variants have non-zero weights) whereas elastic net yields sparse models, setting the weights of most variants to 0. Given the high polygenicity of brain features, we expected ridge regression to perform better than the sparsity-inducing elastic net penalty (Methods).

To evaluate prediction performance, we calculated the Spearman correlation between the observed and predicted brain features with a five-fold cross-validation scheme (Methods), Fig. 3C. For our purposes, the correlation with the observed trait (as opposed to mean squared difference) is a relevant measure of performance. Because we are interested in the association between genetically predicted traits (i.e., IDPs) and complex traits, we are more interested in optimizing the correlation of predictors with IDPs rather than the accuracy of predicted effect magnitudes.

For the ridge regression, the prediction performance ranged from 0.024 to 0.24 with a median of 0.13. For elastic net, the range was between −0.00035 and 0.28 with a median of 0.068. All the ridge predictors and 93 % of the elastic net predictors showed positive cross-validated Spearman correlations (Table S4), demonstrating the feasibility of genetically predicting brain features.

For most brain features, the ridge regression yielded higher cross-validated performance than elastic net, as we hypothesized given the high polygenicity of the features (See Fig. 3C). To further corroborate that ridge regression performs better for more polygenic traits, we plotted the gain in performance by ridge regression over elastic net predictors against the estimated polygenicity. Unsurprisingly, we found that the gain increased with the degree of polygenicity of the brain feature (Fig. S5).

Also, the prediction performance increased as the brain feature heritability increased (Fig. 3D). However, we noted that the median prediction $R^{2}$ was less than 8 % of the heritability, an upper bound for the prediction. The low proportion of heritability captured by the genetic predictors highlights the need to increase the sample size of reference image data to reach the upper bound of the performance.

To reduce false positives in the BrainXcan association analysis, we filtered out unreliable predictors by keeping only brain features that showed prediction performance correlation greater than 0.1. Among structural features, 105 ridge predictors and 54 elastic net predictors passed the threshold from a total of 159 trained. Among 300 diffusion features, 210 ridge predictors and 76 elastic net predictors passed the threshold (limiting to the 192 main diffusion features, 148 ridge and 62 elastic net predictors passed the threshold). All subtype-level PCs, except the elastic net-based PC predictor of cortical region volumes, were well-predicted and retained for the subsequent analysis.

### 2.3 Association module

#### 2.3.1 Application of BrainXcan association module to complex traits

We selected nine traits from the UK Biobank and performed BrainXcan association using 327,743 individuals of British ancestry. As described in the overview section, we calculated the genetically predicted brain features for all individuals and correlated them with the traits using linear regression. Logistic or other generalized linear models can be used for binary and other types of traits. The traits included alcohol consumption, smoking, coffee consumption, depression, parental depression, parental Alzheimer’s disease, handedness, BMI, and height (see detailed list in Table S5). To avoid overfitting issues, we excluded individuals used to train the prediction models.

To assess the sensitivity of BrainXcan to the prediction approach, we compared the BrainXcan Z-scores obtained by using ridge vs elastic net-based predictors. Reassuringly, we found a high concordance between ridge and elastic net results. As expected, ridge predictors yielded higher significance given their increased prediction performance relative to elastic net (fig. S7). We focus on ridge regression-based results for the remainder of our analyses.

#### 2.3.2 S-BrainXcan uses GWAS summary statistics to compute associations

Next, we extended the BrainXcan association module so that it could infer the association statistics from the GWAS summary statistics without the use of individual-level genotype and trait data. We term this version S-BrainXcan. Our approach is similar to the S-PrediXcan method and other summary-based TWAS approaches used for correlating genetically predicted transcriptomes with complex traits (Gusev et al., 2016; Barbeira et al., 2018). However, unlike gene expression prediction, which only uses variants in the vicinity of the gene, IDP prediction requires a much larger number of genetic predictors distributed throughout the genome, presenting a significant computational challenge. To make IDP prediction and association feasible, we developed a scalable method that can handle this added complexity (Methods). Specifically, we calculated the covariances between the SNPs used in the prediction and saved them in a sparse format, setting the correlations between distant SNPs (i.e. separated by more than 200 SNPs) to be 0. We chose a separation of 200 SNPs because it allows for reasonably sized reference files without significantly compromising the accuracy of the covariances. We used the HapMap3 subset of SNPs for the prediction in anticipation of this computational burden.

We applied S-BrainXcan association analysis on 35 traits for which we had access to GWAS summary statistics, but not individual-level data. These traits included behavioral, psychiatric and neurologic traits, height, and body mass index (see Table S6). The full results of the S-BrainXcan associations for these 35 GWASs are available in Table S7.

**Fig. 4:**
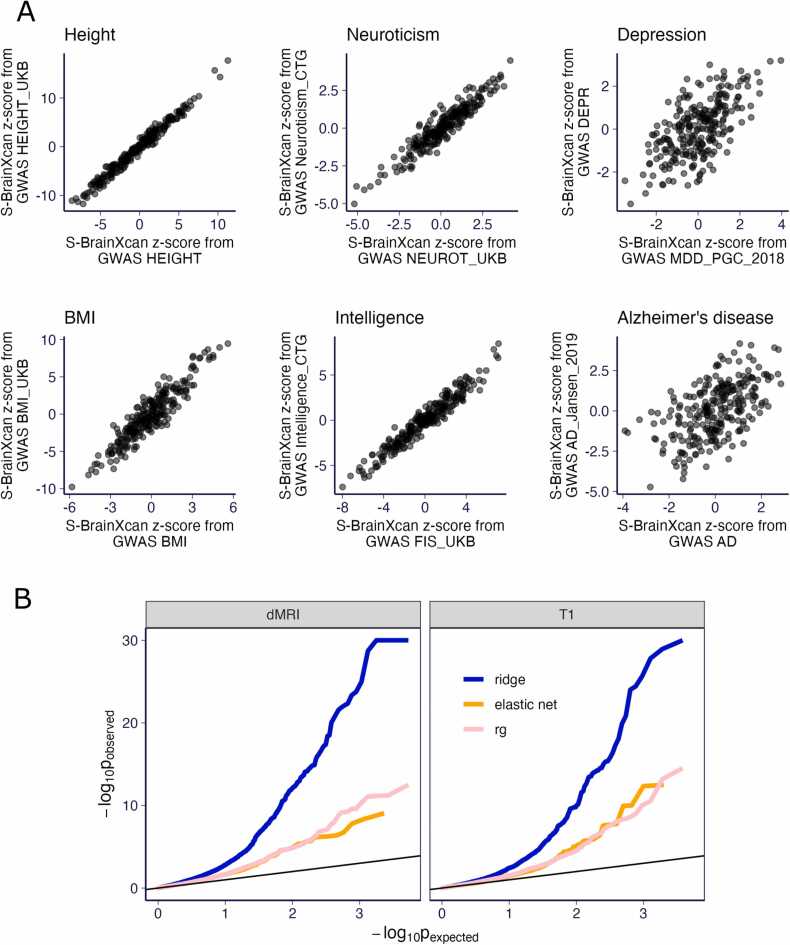
S-BrainXcan association statistics for selected GWAS. A) Six traits which have multiple GWAS were analyzed by S-BrainXcan are shown. For each brain feature, the S-BrainXcan Z-scores (from ridge models) from the two GWAS are shown on x-axis and y-axis respectively (see the GWAS label in Table S6). B) The S-BrainXcan p-values for all 35 GWASs are shown as a QQ-plot (against the expected p-values under the null). For visualization purposes, the observed p-values smaller than $1\times 10^{-30}$ are set to $1\times 10^{-30}$. Label ‘rg’ represents the genetic correlation result. The black lines are the identity line (y=x).

For standing height and BMI, we confirmed the reliability of the S-BrainXcan by comparing the Z-scores of the associations to the ones obtained from individual-level BrainXcan using data from the UK Biobank. Overall, Z-scores were highly concordant, reassuring us that the approximations used in the implementation of the summary version of BrainXcan did not have a detrimental effect on the results (Fig. S6). We also compared S-BrainXcan Z-scores from independent studies for height, neuroticism, depression, BMI, intelligence, and Alzheimer’s disease. Reassuringly, we found highly concordant Z-scores between the independent studies (Fig. 4A).

Among those traits analyzed by S-BrainXcan, 177 out of the 261 of tested brain features were significantly associated with at least one trait (Bonferroni p-value cutoff = 0.05 / 261 for each trait). As expected, better powered GWAS traits with a larger number of significant associations also yielded more significant feature-to-trait associations.

Brain-wide features tended to be more significantly associated than region-specific features, suggesting a more prominent role in complex traits (Fig. S8). However, the increased significance of the brain-wide features could be attributed to their better predictive performance, which suggests they are less noisy and therefore less influenced by attenuation bias (Supplementary Notes 1).

#### 2.3.3 Addressing inflation of type I error

As reported in Liang et al. (2023), association between genetically predicted traits and polygenic complex traits can yield significance levels that are not well calibrated when the trait is highly polygenic and the GWAS sample sizes are largeconditions which apply to BrainXcan. Briefly, Liang et al. showed that the expected $\chi^{2}$ statistic (i.e., the expected square of the Z-score of the association) is greater than 1 under the null, which leads to inflated significance if not properly corrected. They showed that

$$EZ^{2}\approx 1+Nh^{2}\Phi ,$$

where N is the sample size of the GWAS, $h^{2}$ is the heritability of the trait Y, and Φ is a factor specific to each IDP that can be pre-calculated using multiple simulations of polygenic null phenotypes and their associations with the actual predicted IDPs. Additional details on the calculation of Φ are in the Methods section. We applied this correction to all reported BrainXcan results. The end user of the BrainXcan software will have the corrected p-values in the standard output so no additional correction will be necessary.

#### 2.3.4 Genetic correlations yield similar, but less significant, associations

Genetic correlations between brain features and complex traits can provide, in principle, similar information to BrainXcan association results. We computed the correlations for all featuretrait pairs and compared the results to BrainXcan associations. (Methods; Fig. 4B). The full results of the genetic correlations for these 35 GWASs are available in Table S8. We found that the Z-scores of the genetic correlations were highly correlated to the Z-scores of BrainXcan associations (with correlations ranging from 0.51 to 0.97, with a median of 0.81; Fig. S9). However, BrainXcan yielded a 3-fold increase in significant featuretrait associations compared to the genetic correlation approach, suggesting that using optimal predictors of brain features has more power to identify putative causal links.

#### 2.3.5 Mendelian randomization module

The association module of BrainXcan cannot directly address the direction of causality (i.e., if genetically predicted brain features are the cause or result of the disease state). Therefore, we implemented the Mendialiam randomization module to evaluate the causal relationship of each association. Mendelian randomization evaluates the causal relationship between traits by testing whether increased "exposure" to the first trait (represented as the trait 1 GWAS effect size) is associated with an increased or decreased level of the second trait (indicated by the trait 2 GWAS effect size). In Mendelian randomization settings, individuals are thought to be randomized at meiosis to either inherit the risk-increasing allele or not. Because of these parallel to randomized trials, the level of causal evidence derived from Mendelian randomization is considered to be higher than that of observational studies, but lower than that of actual randomized trials (the gold standard for causal determination used in clinical trials).

It is possible to infer the direction of the causal flow by selecting genetic variants that have strong effects on the first trait and testing for a significant association with the effect sizes of the second trait and vice versa (Pickrell et al., 2016). As described below, scatter plots of effect sizes showing the two selection strategies (by significance of trait 1 or 2), are added to the automated reports.

We applied several Mendelian randomization approaches, including MR-BASE (Hemani et al., 2018), inverse variance weighted regression (Burgess et al., 2013), weighted median method (Bowden et al., 2016) and Egger regression (Bowden et al., 2015), to determine the direction of the putative causal flow. In all analyses, we assumed that multiple genetic variants are strongly associated with the phenotype (we refer to these as variants as instruments). For complex traits, we selected SNPs with GWAS p-values $<5\times 10^{-8}$ and for IDPs, we selected SNPs with GWAS p-values $<10^{-5}$. The less stringent p-value for IDPs was necessary to have a sufficient number of instruments, which can be adjusted as the number of individuals in the IDP dataset increases. To streamline the interpretations of the multiple Mendelian randomization results, we combined the p-values of each Mendelian randomization output using an extension of the ACAT method (Liu et al., 2019), which accounts for concordance of the sign of the results (Supplementary Notes 3). Results for all Mendelian Randomization approaches are provided with the output of the software. See caveats for interpreting the Mendelian Randomization results in Discussion and Supplementary Notes 4 and Fig. S11.

#### 2.3.6 Application of BrainXcan to schizophrenia risk

Brain imaging data has been widely utilized for deciphering the link between brain microstructure and schizophrenia biology (Van Erp et al., 2018; Simões et al., 2020; Zhao et al., 2021; Stauffer et al., 2021). To demonstrate the features of BrainXcan, we applied the full BrainXcan pipeline to a schizophrenia GWAS (Ripke et al., 2020). We focused on 261 features, which include 48 cortical gray matter volumes, 10 subcortical volumes, 13 subcortical gray matter volumes, 27 cerebellum gray matter volumes, fractional anisotropy in 46 regions, intracellular volume fraction (ICVF) in 44 regions, and isotropic volume fraction (ISOVF) in 13 regions. (We note that features in the ISOVF category were less heritable leading to fewer successful predictors). We also included brain-wide measures represented by leading principal components of each subtype (gray volumes of cortical, cerebellum, and subcortical regions, subcortical total volumes, FA, ICVF, OD, and ISOVF).

**Fig. 5:**
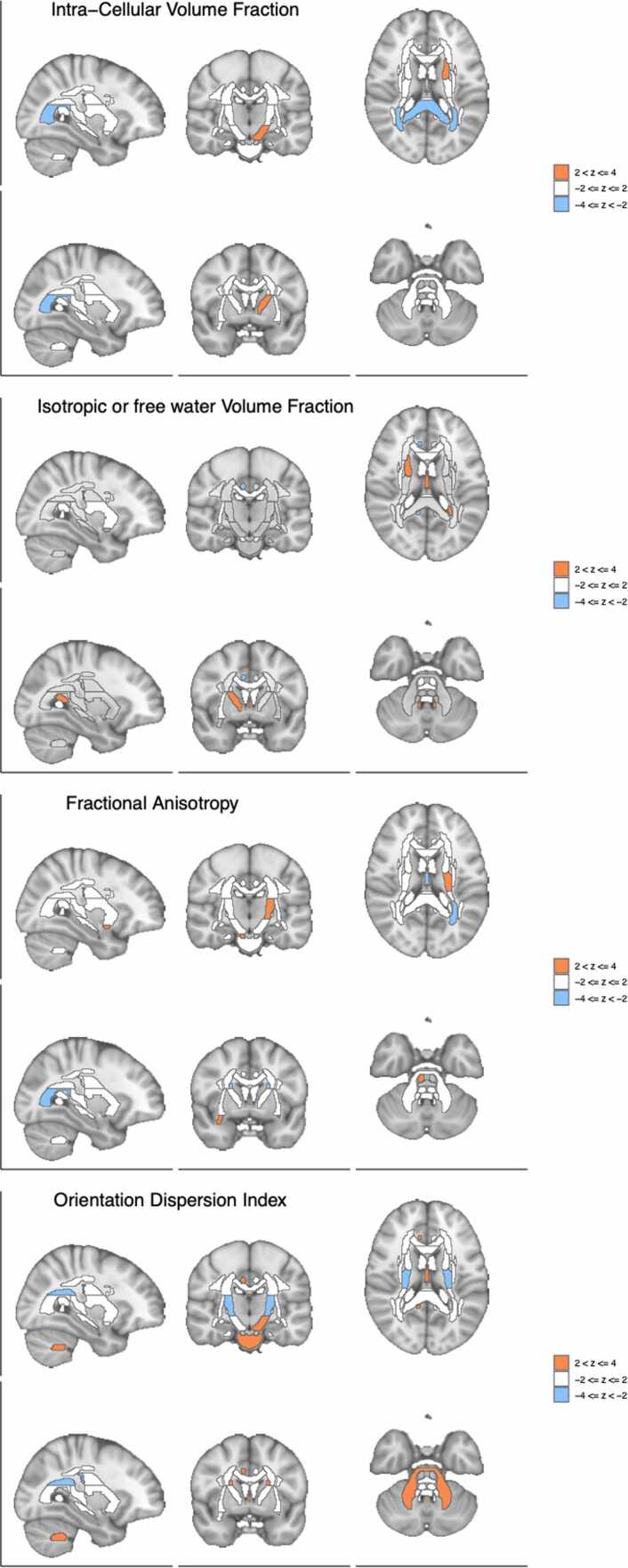
Brain visualization of diffusion features associations with schizophrenia risk. Z-scores of the associations between the brain region and schizophrenia risk are shown with different slices of the brain.

**Fig. 6:**
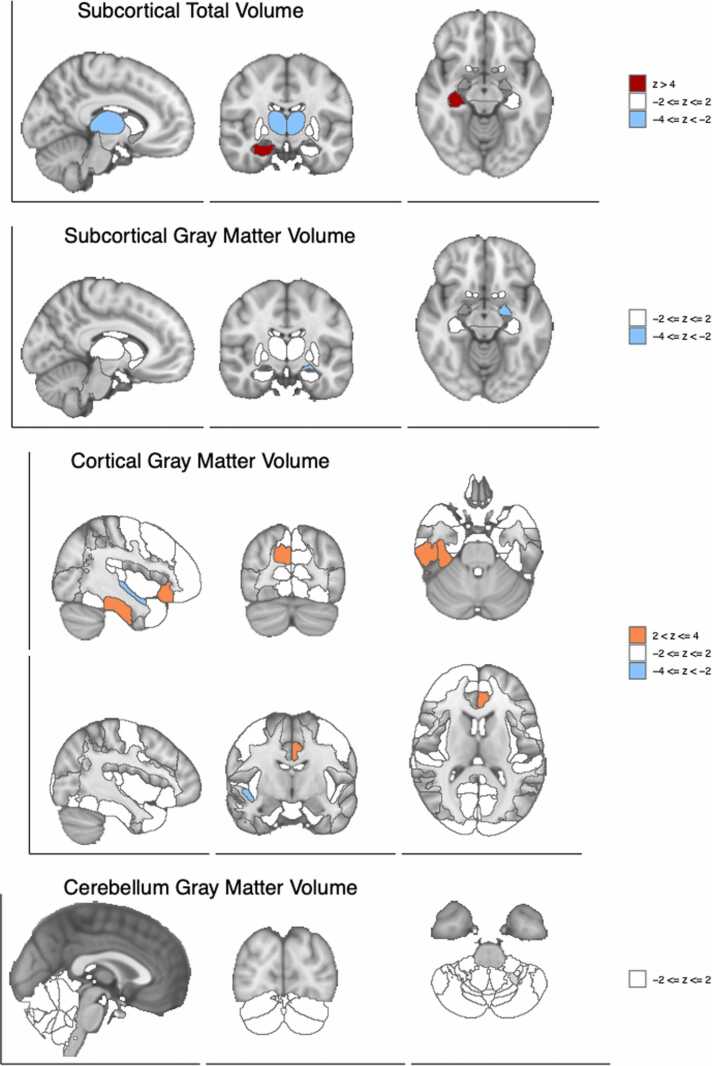
Brain visualization of structural features associations with schizophrenia risk. Z-scores of the associations between the brain region and schizophrenia risk are shown with different slices of the brain.

Among the 261 features, three were significantly associated with risk of schizophrenia (FDR < 10 %). Fig. 5, Fig. 6 provide a snapshot for schizophrenia risk. To aid interpretation, an interactive annotation of different regions of the brain is added to the output of the software’s automated pipeline (See example at https://hakyimlab.github.io/brainxcan-docs/docs/report.html).

Among diffusion MRI associations, the principal component of ICVF, a proxy for brain-wide axonal density, was significantly associated with schizophrenia risk, with lower axonal density associated with higher risk of schizophrenia (Fig. S10). The first principal component of fractional anisotropy, a brain-wide measure of water diffusion efficiency along nerve tracts, was also negatively associated with schizophrenia, while the OD index (dispersion of neurite orientation along tracts) showed no significant association with schizophrenia.

The total volume of the hippocampus (right side, relative to brain size) was positively associated with schizophrenia risk, which has been reported to be associated with schizophrenia status (van Erp et al., 2016; Shepherd et al., 2012).

Despite the limitations of Mendelian Randomization analysis in the context of high polygenicity (see Supplementary Notes 4), we conducted the analysis because the results can provide suggestive evidence and help generate hypotheses. Among the top 10 features associated with schizophrenia, we identified three instances of feature-to-schizophrenia risk causal flow with nominally significant p-values (p < 0.05), and six instances in the opposite direction. The feature-to-schizophrenia results suggest a potential mechanism for schizophrenia risk, even though the highly polygenic nature of IDPs limits the power of Mendelian Randomization methods, which require strong instruments (i.e., highly significantly associated variants). However, the schizophrenia-to-feature results should not be interpreted as indicating an effect of schizophrenia on the image feature (scenario B in Fig. S11). In fact, an effect of the image feature on schizophrenia can be ruled out, as our training samples did not include schizophrenia cases. Therefore, the genetically predicted image feature does not reflect a consequence of schizophrenia. As discussed in the supplementary note, given the power differential between the schizophrenia GWAS and the image feature traits, we cannot rule out scenarios A and C. Scenario A corresponds to the image feature affecting schizophrenia, and scenario C corresponds to genetic variants affecting an unobserved feature that influences both schizophrenia risk and the image feature.

## 3 Discussion

We present a robust, scalable framework we call BrainXcan that leverages genetically predicted brain features trained on reference MRI datasets, GWAS of complex diseases, and computational and methodological advances to dissect the biology of behavioral, neurological, and psychiatric traits. Our approach addresses the sample size limitations of MRI studies by taking advantage of increasing cohorts of GWAS studies and large MRI data in predominantly healthy subjects. The use of genetic variation helps us circumvent the reverse causality problem.

Our association module identifies brain features likely to influence behavioral and psychiatric traits and/or features that can be modified by the disease. Our Mendelian randomization module quantifies the evidence for both directions of the putative causal flow (brain feature-to-disease or vice-versa). Naturally, both directions of the effects are informative. Understanding how human disease causes changes in brain features captured by MRI can help design better diagnostic tools. Brain features that modulate disease risk provide insights into disease pathogenesis and can help identify preventive or therapeutic strategies.

Similar analyses can be performed using genetic correlation or colocalization approaches, but they tend to be much less powerful than a direct association approach as implemented in BrainXcan. For instance, colocalization methods have been shown to have high false negative rates (Barbeira et al., 2021). We suggest performing these alternative analyses to get additional lines of evidence, which, in teh case of concordance, should strengthen confidence in results obtained with BrainXcan.

To encourage broad adoption of BrainXcan by users less familiar with genetic tools, we provide a user-friendly pipeline and an automated report. All the tools necessary to perform prediction, association (including inflation correction), and causal flow assessment are provided (https://github.com/hakyimlab/brainxcan).

In the process of developing BrainXcan, we learned that, similar to psychiatric and behavioral traits, brain features are highly polygenicin many cases even more so than human height, which is the canonical example of a polygenic trait. Their shared similarity in genetic architecture suggests that brain features can be useful endo-phenotypes to improve the classification of complex psychiatric diseases.

We present an application to schizophrenia to showcase the potential of our method. The significant association between low levels of brain-wide ICVF (a proxy for axonal density) with high risk of schizophrenia corroborates the long standing hypothesis that schizophrenia is a disorder of disconnectivity. Our results are also consistent with reduced FA in schizophrenia cases compared to controls as reported by Kelly et al. (2018). Since our technique uses healthy individuals for MRI-based genetic prediction of brain features, it is less likely to capture features that are a consequence of the disease.

We note that there are several limitations in the current work. First, the prediction performance of the current genetic predictors are largely limited by the size of the training cohort (Fig. 3d). As the UK Biobank is gradually collecting more brain imaging data (Littlejohns et al., 2020), we expect that the training cohort size will increase to at least 100,000 individuals within the next few years. We will be updating the brain feature predictors as more data becomes available. Second, the S-BrainXcan calculation relies on the genotype covariance, which is approximated as a banded matrix (Methods). This approximation may affect the stability of a joint analysis (with multiple brain features) which relies heavily on the predicted feature covariance derived from the genotype covariance. We decided not to pursue a joint model to avoid false positives driven by LD-misspecification. Third, the BrainXcan analysis cannot establish a causal relationship between the brain features and the complex trait. Although we run Mendelian randomization in both the forward and the reverse directions for the featuretrait candidate, the results should be interpreted with caution due to the following reasons: i) different Mendelian randomization tests may not give consistently significant results; ii) the Mendelian randomization of the forward and reverse directions typically have different power due to differences in sample sizes and genetic architecture; iii) if the same GWAS is used for both BrainXcan association and Mendelian randomization, the Mendelian randomization p-value is not well-calibrated; iv) causality is valid only when the Mendelian randomization assumptions are held; and v) due to the high polygenicity and small effect sizes of IDPs, our results suffer from weak instrument bias. Given these limitations, the Mendelian randomization results should not be interpreted as definitive support for the direction of the causal flow. Increased reference image datasets and additional model development that account for these limitations are needed. Nevertheless, we think that the Mendelian randomization analysis offers valuable additional insights. Fourth, only linear prediction models are used in our implementation. Although more sophisticated models could be used for prediction, this would limit the application to cases where the full individual-level data is available. However, given the difficulties of accessing and handling individual-level data at a very large scale, a linear model seems reasonable. Fifth, we do not account for uncertainty in this study. However, given the attenuation bias this will cause, our result is conservative. Future improvements in prediction models will generally mitigate this issue. Sixth, the training of the prediction models was performed in European samples which likely limits the performance in other populations. As more diverse data are available, we will use them to improve portability of prediction models across populations. Despite these limitations, we anticipate that BrainXcan, a user-friendly analysis tool, will help identify brain features important in the pathogenesis as well as diagnosis of complex traits.

## 4 Methods

### 4.1 Preprocessing of UK Biobank brain features

We queried the UK Biobank database using ukbREST (Pividori and Im, 2019) to retrieve the list of 459 MRI image-derived phenotypes (IDPs, also referred to as brain features or features) as shown in Table S2 (Smith et al., 2020). Among the features, 300 were derived from diffusion MRI and 159 from T1-weighted structural measurements. For the diffusion features, we focused our analysis on the 192 features mapped to the Johns Hopkins University’s 48 region atlas tracts (Wakana et al., 2004). The T1-weighted structural measurements include 139 FAST-based grey matter segmentation-based measurements and 14 FIRST-based subcortical structures measurements and 6 T1 structural brain MRIs measuring the total volumes of the peripheral cortical grey matter, ventricular cerebrospinal fluid, brain grey matter, brain white matter, brain grey and white matter, and brain stem. See technical details in Miller et al. (2016). In total, we collected 24,409 European-descent individuals in UK Biobank with non-missing brain features and non-missing values for other covariates, such as genetic PCs, sex, and age at recruitment.

We scaled the structural features using the volumetric scaling factor from the T1 head image (UK Biobank Data-Field 25000) so that the measurement of the brain region volume was relative to the total brain volume. Additionally, we regressed out the following scanner position covariates: UK Biobank data fields 25756 (Scanner lateral (X) brain position), 25757 (Scanner transverse (Y) brain position), 25758 (Scanner longitudinal (Z) brain position), and 25759 (Scanner table position). We also regressed out the first 10 genetic PCs, age, sex, age squared, age × sex, and age squared × sex.

To adjust for the correlation between features and to extract the factor in common across different regions of the brain, we performed principal component analysis on the IDP matrices (individual-by-IDP matrices) within each IDP subtype. For T1 IDPs, the subtypes were gray matter volume of the cortical regions, gray matter volume of the subcortical regions, total volume of subcortical regions, and gray matter of the cerebellum regions. For diffusion IDPs, the subtypes were defined by the four measure types (FA, ICVF, ISOVF, and OD). We consider the first PC to represent the common factor for each IDP subtype. To define the region-specific components, we calculated the residuals after regressing out the first PC from the IDPs (see discussion in Supplementary Notes 2 and fig. S12). To improve robustness and reduce the influence of outliers, we inverse-normalized the PCs and the residuals.

### 4.2 Selecting genetic variants from UK Biobank imputed genotypes

To reduce the computational burden, we restricted the prediction to the HapMap 3 subset of variants with minor allele frequency above 1 % among CEU (European-descent) individuals (International HapMap 3 Consortium and others, 2010). Among these variants, we kept variants with MAF > 5 % in UKB IDP cohort (all of European-descent). We also excluded ambiguous variants which have reverse complementary bases as the reference and the alternative alleles (AT and CG pairs). In total, 1,078,323 variants passed our criteria and among these, 1,071,649 variants were present in the UK Biobank imputed genotype data (by SNP rsID). In the subsequent analysis, we limited the computation to this set of variants.

### 4.3 Estimating the heritability

We estimated the heritability of IDPs assuming random effects for SNPs (Yang et al., 2010). We used the EMMA algorithm proposed in Kang et al. (2008) to avoid repeated calculations when dealing with multiple phenotypes from the same cohort. In the analysis, the genetic relatedness matrix (GRM) was built using the pre-selected HapMap 3 SNPs. Since we regressed out MRI technical covariates, the first 10 genetic PCs, age, sex, squared age, age × sex, and squared age × sex in the pre-processing step, we only added the intercept as a covariate in the heritability calculation.

### 4.4 Estimating of polygenicity

To quantify polygenicity, we estimated the effective number of independently associated SNPs using the stratified LD fourth moments regression method (O’Connor et al., 2019). We downloaded the pre-computed LD scores and LD fourth moments from https://www.dropbox.com/sh/iiyftw01gdpt6un/AACU7AmWK45RxTmDJvRkdKhIa?dl= 0 and used the scores stored in baselineLD.1 kg.l2l4.mat. These scores were based on baselineLD annotations (Gazal et al., 2017). The stratified LD fourth moments regression was performed in MATLAB by calling SLD4M function shared in https://github.com/lukejoconnor/SLD4M (O’Connor et al., 2019). To obtain the effective number of independently associated common SNPs, we aggregated the estimated $M_{e}$ values across 10 MAF bins which correspond to the common variants (MAF > 0.05). The aggregation was done by setting report_annot_mat variable in the SLD4M function.

### 4.5 Building polygenic predictors of brain features

We built elastic net and ridge regression predictors of brain features, one feature at a time. As mentioned above, the prediction used the pre-selected 1,078,323 common HapMap3 variants. Since we already adjusted for covariates, we included only the intercept as a covariate in the model training.

### 4.6 Ridge regression models

For ridge regression we performed the following optimization problem:

$$\arg\min_{w}∥B-\mathrm{Xw}{∥}_{2}^{2}+\lambda ∥w{∥}_{2}^{2},\;(1)\;$$

where B is n×1 the mean-centered brain feature (fitting one at a time), X is the n×P standardized genotype matrix (mean centered and divided by the standard deviation) of the variants, and w is the P×1 prediction weights. Since the number of variants (P) is much larger than the number of samples (n), instead of using the usual ridge regression solution

$${\hat{w}}^{\mathrm{ridge}}={\left(X^{\prime }X+\lambda I_{P}\right)}^{-1}X^{\prime }B,$$

we use the equivalent form

$${\hat{w}}^{\mathrm{ridge}}=X^{\prime }{\left(XX^{\prime }+\lambda I_{n}\right)}^{-1}B,$$

which can be derived using the ‘matrix push through identity’. λ is a hyperparameter to be determined. $I_{P}$ and $I_{n}$ are P×P and n×n identity matrices.

Let Σ represent the GRM matrix and we have $\Sigma =XX^{\prime }∕P$, where X is the genotype matrix in the training set. The expression for the w^ridge can be re-parameterized with $\theta =\frac{P}{P+\lambda }$ which takes value in the 0–1 range.

$${\hat{w}}^{\mathrm{ridge}}=\theta X^{\prime }{\left(\theta \Sigma +(1-\theta )I_{n}\right)}^{-1}B.$$

We chose the optimal hyperparameter θ by performing 5-fold cross-validation on a grid of θ values 0.01, 0.05, 0.1, 0.2, 0.3, 0.4, 0.5, 0.6, 0.7, 0.8, 0.9, 0.95. We also implemented a training scheme with a splitting of 80 % data for training and 20 % data for validation to compare directly to elastic net models (see details below) and observed no significant difference in terms of the performance of the two training schemes (cross-validation or 80 %/20 % splitting) for ridge regression models.

### 4.7 Elastic net models

We trained the elastic net predictors using the R package snpnet which implements the BSAIL algorithm proposed by Qian et al. (2020). Specifically, we fit the following optimization problem:

$$\arg\min_{w}\frac{1}{2N}∥B-\mathrm{Xw}{∥}_{2}^{2}+\lambda (\frac{1-\alpha }{2}∥w{∥}_{2}^{2}+\alpha ∥w{∥}_{1})\;(2)\;$$

We set the mixing hyperparameter α to 0.1. To choose the optimal hyperparameter λ, we divided the brain reference data into a training set with 80 % of the samples and a validation set with the remaining 20 % of the samples. We performed elastic net regression using a grid of λ values determined by snpnet (default parameters were used) and selected the λ that yielded the largest correlation between predicted and actual B in the validation set. Finally, we trained the final prediction model using the full set of data (training and validation). When using snpnet, we set the maximum number of iterations (niter) to 100 and the number of SNPs to consider in each batch (num.snps.batch) to 200. Each brain feature was trained separately.

### 4.8 Calculating the prediction performance

For both ridge and elastic net models, we evaluated the prediction performance by 5-fold cross validation. For each fold, we used the remaining 4 folds to train the prediction models using the procedures described above. The prediction weights were used to predict on the held-out fold of the data. This procedure was repeated for all five splits and the predicted values were aggregated across all folds. The prediction accuracy was evaluated in terms of $R^{2}$, Pearson correlation, and Spearman correlation.

### 4.9 BrainXcan with summary statistics

When the individual-level information was not available, we calculated the BrainXcan statistic approximately using the GWAS summary statistic and the genotype covariance from a reference panel.

The formulas are similar to Gusev et al. (2016) and Barbeira et al. (2018). Briefly, let ${\hat{b}}_{j}$ and $\mathrm{se}({\hat{b}}_{j})$ be the estimated effect size and the corresponding standard error for variant j from the GWAS. And $z_{\text{GWAS},j}$ is the GWAS Z-score of SNP j. Let $\hat{R}$ represent the genotype sample covariance matrix where ${\hat{R}}_{jj^{\prime }}=\hat{\mathrm{Cov}}(X_{j},X_{j}^{\prime })$, namely the sample covariance between variant j and $j^{\prime }$. We can calculate the marginal test statistics using the following results:

$${\hat{\beta }}_{k}=\frac{\sum_{j=1}^{P}{\hat{w}}_{\mathrm{kj}}{\hat{R}}_{\mathrm{jj}}{\hat{b}}_{j}}{{\hat{\sigma }}_{k}^{2}}\;(3)\;$$

$z_{\text{BrainXcan},k}\;\approx \frac{1}{{\hat{\sigma }}_{k}}\;\sum_{j=1}^{P}{\hat{w}}_{\mathrm{kj}}\sqrt{{\hat{R}}_{\mathrm{jj}}}\;z_{\text{gwas},j}\;(4)$

$${\hat{\sigma }}_{k}^{2}\;=\sum_{j=1}^{P}\sum_{j=1}^{P}{\hat{w}}_{\mathrm{kj}}{\hat{w}}_{\mathrm{kj}\prime }\;{\hat{R}}_{\mathrm{jj}\prime }\;,\;(5)$$

where $z_{\text{BrainXcan},k}$ represents the BrainXcan marginal test Z-score for the kth brain IDP. We refer this summary statistic-based BrainXcan as S-BrainXcan.

In principle, we need to consider the genotype covariance for all the genome-wide variant pairs. To reduce the computational burden, we first assumed that the between-chromosome covariance is zero. Moreover, we considered the per-chromosome genotype covariance matrix as a banded matrix with bandwidth equal to 200. In other words, any variant pairs with more than 200 variants in between are considered to have zero covariance. Results remain substantively similar when using larger number of SNPs, up to 1000. As shown in the results, the concordance between summary- and individual-level based results reassured us that this was a reasonable trade-off to keep the total size of covariance files that the user needs to download below 50 GB. We used the set of 24,409 UK Biobank individuals included in the brain IDP model training as the reference panel for genotype covariance calculation.

### 4.10 Computing inflation factor Φ

To estimate the inflation factor, we followed the procedure described in Liang et al. (2023). We used genotype of unrelated individuals from the UK biobank. We used the HapMap3 subset of SNPs since our prediction models only used these variants. We computed the genetic predictors of IDPs using the ridge regression weights we generated. We simulated a null phenotype $Y=\sum_{k}X_{k}\delta_{k}+\epsilon$ with normally distributed effect sizes $\delta_{k}$ for a range of sample sizes - N (ranging from 1000 to 20,000) and heritability values - $h^{2}$(ranging from 0.01 to 0.99).

For each of the combination of sample size, heritability and IDP, we ran a linear regression of the null phenotype against the predicted IDP to estimate the IDP effect on the phenotype Y. We repeated the regression for 1000 simulations and calculated the average χ2. Using the average $\chi^{2}$ we estimated the inflation factor of each IDP by fitting a mixed-effects model with a common intercept for all IDPs and a IDP-specific random effect.

Once we calculated the inflation factor for every IDP, we saved them in the prediction model database with the weights. The correction is performed by calculating the p-values using the variance control method: pchisq(chi2/sqrt(1 + N * h2 * Phi)). This correction is implemented in the BrainXcan software.

### 4.11 Performing GWAS for brain features

We performed GWAS for all the brain IDPs using the Python package tensorqtl (Taylor-Weiner et al., 2019). Since we regressed out MRI technical covariates, the first 10 genetic PCs, age, sex, squared age, age × sex, and squared age × sex in the pre-processing step, the intercept was the only covariate included in the tensorqtl runs.

### 4.12 Mendelian randomization analysis of IDP/phenotype pairs

To investigate the direction of the effect, we performed Mendelian randomization (MR) analysis for the significant featuretrait pairs identified in the BrainXcan association stage. The MR analysis was performed for both directions: i) brain feature → complex trait, treating the brain feature as the mediating phenotype and the trait of interest as the outcome trait; ii) trait → brain feature, the trait of interest as the mediating phenotype and the brain feature as the outcome trait. As the inputs of the analysis, we used the brain IDP GWAS results as described above. For the phenotype of interest, we used the GWAS results, which were also used for the S-BrainXcan analysis. The MR analysis was performed using the R package TwoSampleMR (Hemani et al., 2018).

For the mediating trait, the instrument variants were selected using the LD clumping function (ld_clump) in the R package ieugwasr (Elsworth et al., 2020). We used the EUR super-population in 1000 Genomes data (1000 1000 1000 Genomes Project Consortium, 2015) as the LD reference panel, which we downloaded from http://fileserve.mrcieu.ac.uk/ld/1kg.v3.tgz. The LD clumping parameters were clump_kb = 10,000 and clump_r2 = 0.001. The p-value parameter (clump_p) in the LD clumping was $10^{-5}$ for IDP GWAS and $5\times 10^{-8}$ for phenotype GWAS, which gave approximately independent and significant variant instruments.

We reported the MR results using three MR methods: i) inverse variance weighted MR (Burgess et al., 2013); ii) median-based estimator: weighted median (Bowden et al., 2016); iii) MR Egger analysis (Bowden et al., 2015), which corresponds to mr_ivw, mr_weighted_median, and mr_egger_regression in TwoSampleMR. We further reported a meta-analyzed p-value summarizing the results of the three MR tests being performed. The meta-analysis is based on an extension of ACAT method (Liu et al., 2019) that takes into account the direction of the effects. See Supplementary Notes 3 and Fig. S13 for additional details.

### 4.13 Calculating the genetic correlation for brain feature/trait pairs

The genetic correlation between a brain feature and the trait of interest was calculated using the cross-trait LD Score regression (Bulik-Sullivan et al., 2015) implemented in the Python package ldsc (https://github.com/bulik/ldsc). The pre-computed LD-scores were downloaded from https://storage.googleapis.com/broad-alkesgroup-public/LDSCORE/eur_w_ld_chr.tar.bz2, which are based on the 1000 Genomes European data. We used the brain feature GWAS results as described above and the GWAS data for each phenotype was the same as the data used for the S-BrainXcan analysis.

## CRediT authorship contribution statement

**Brown Andrew:** Writing – review & editing, Investigation. **Im Hae Kyung:** Writing – review & editing, Writing – original draft, Supervision, Methodology, Funding acquisition, Formal analysis, Conceptualization. **Liang Yanyu:** Writing – review & editing, Writing – original draft, Visualization, Software, Methodology, Formal analysis, Data curation, Conceptualization. **Nyasimi Festus:** Writing – review & editing, Visualization, Formal analysis. **Melia Owen:** Writing – review & editing, Methodology, Data curation. **Carroll Timothy J:** Writing – review & editing, Investigation. **Brettin Thomas S:** Writing – review & editing, Investigation.

## Declaration of Competing Interest

The authors declare that they have no known competing financial interests or personal relationships that could have appeared to influence the work reported in this paper.

## Data Availability

We use publicly available data. Genetic and image derived phenotypes were obtained from the UK Biobank under Application Number 19526 and 89052. GWAS summary statistics were downloaded from the public domain (url in Table S6). BrainXcan software is available at https://github.com/hakyimlab/brainxcan. We are sharing image derived phenotypes prediction models at https://doi.org/10.5281/zenodo.4895174. The analysis code is at https://github.com/liangyy/ukb_idp_genetic_arch. Phi estimation code is at https://github.com/hakyimlab/twas-inflation
