# Supplementary material for: BrainXcan identifies brain features associated with behavioral and psychiatric traits using large-scale genetic and imaging data
Source: Dev Cogn Neurosci. 2025 Mar 13;73:101542. doi: 10.1016/j.dcn.2025.101542 (PMC11964658; doi:10.1016/j.dcn.2025.101542)
Supplement: Supplementary file 1 — Supplementary material [file mmc1.pdf]

Supplementary Materials: BrainXcan identifies brain features associated with behavioral and psychiatric traits using large scale genetic and imaging data

Yanyu Liang[1,*] Festus Nyasimi[1] Owen Melia[2] Timothy J. Caroll[3]
Thomas Brettin[4,5] Andrew Brown[6] Hae Kyung Im[1,4,*]

1 Section of Genetic Medicine, University of Chicago, Chicago, Illinois, United States of America
2 Department of Computer Science, University of Chicago, Chicago, Illinois, United States of America
3 Department of Radiology, University of Chicago, Chicago, Illinois, United States of America
4 Computing Environment and Life Sciences Directorate, Argonne National Laboratory, Argonne, Illinois, United States of America
5 Consortium for Advanced Science and Engineering, University of Chicago, Chicago, Illinois, United States of America
6 Department of Population Health and Genomics, University of Dundee, Dundee, United Kingdom
∗ Correspondence to yanyul@uchicago.edu and haky@uchicago.edu

List of Figures

S1 The first PC of each IDP modality . . . 3
S2 The correlation between IDPs for T1 modalities . . . 4
S3 The correlation between IDPs for dMRI modalities . . . 5
S4 Comparing estimated M_e from [3] and our pipeline . . . 6
S5 Increased performance of ridge predictors vs. polygenicity M_e . . . 7
S6 Comparing individual-level BrainXcan and S-BrainXcan results on UK Biobank standing height and BMI . . . 8
S7 Comparing the ridge and elastic net based individual-level BrainXcan results . . . 9
S8 Comparing the S-BrainXcan significance between region-specific IDPs and common factors . . . 10
S9 Comparing z-scores of the genetic correlation and S-BrainXcan . . . 11
S10 Schizophrenia risk association with diffusion MRI . . . 12
S11 Mendelizan Randomization causal flow interpretation . . . 13
S12 Comparing BrainXcan results from residual IDP and IDP adjusted by PC . . . 14
S13 SACAT based p-value distribution under the global null . . . 15

List of Tables

S1 Demographic information of the IDP cohort . . . 16
S2 UK Biobank brain IDPs being analyzed . . . 16
S3 The heritability and polygenicity estimates of brain IDPs . . . 16
S4 The prediction performance of the ridge and elastic net predictors . . . 16
S5 The list of 9 UK Biobank based phenotypes being analyzed by individual-level BrainXcan . . . 17

S6 The list of 35 GWASs being analyzed by S-BrainXcan . . . 17
S7 S-BrainXcan results for 35 GWASs . . . 17
S8 LDSC based genetic correlation results for 35 GWASs . . . 17

Contents of Supplementary Notes

1 Deriving bias of BrainXcan estimates 18
1.1 A generative model of IDP–phenotype association . . . 18
1.2 Variances and covariances among variables . . . 19
1.3 Biases of the BrainXcan associations . . . 19

2 Using IDP residual instead of fitting IDP and PC jointly 20

3 Aggregating Mendelian Randomization test results by extending the Aggregated Cauchy Association test (ACAT) method 21

4 Caveats on interpreting Mendelian randomization results 23

Supplementary Figures

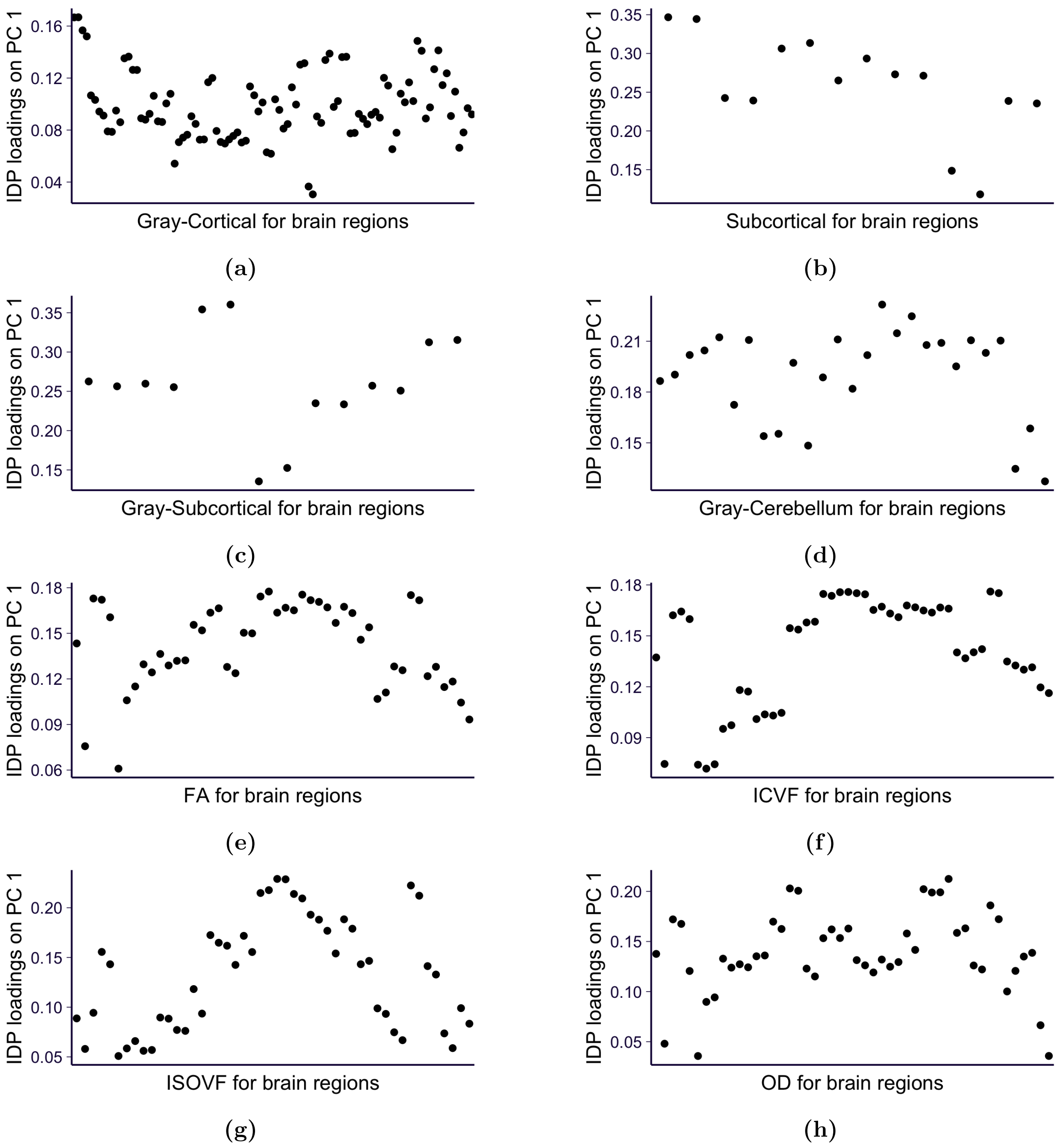

fig. S1. The first PC of each IDP modality For each IDP modality, the contribution of PC1 to each of the brain IDPs within the modality group is shown. Panel **a)** to **d)** show results for T1 modalities: gray matter volume of cortical regions, total volume of subcortical regions, gray matter volume of subcortical regions, and gray matter volume of cerebellum regions. Panel **e)** to **h)** show results for TBSS-based dMRI modalities: FA, ICVF, ISOVF, and OD.

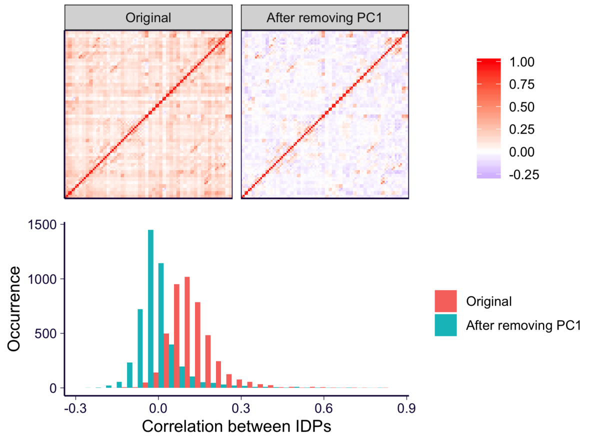

(a)

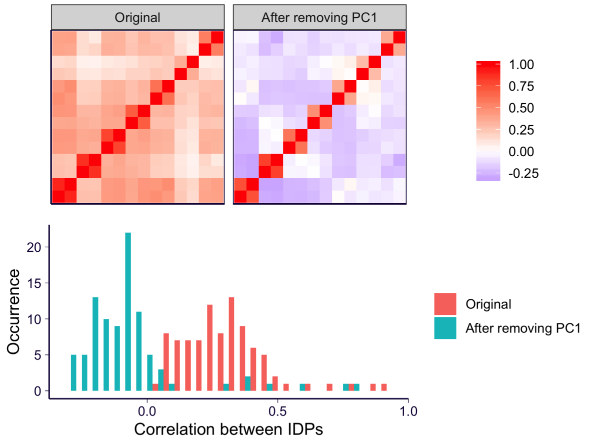

(b)

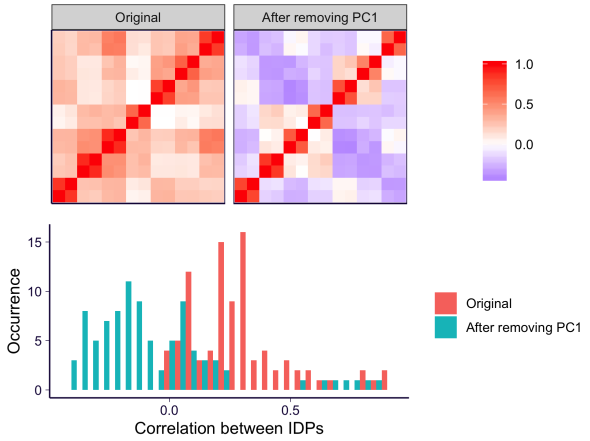

(c)

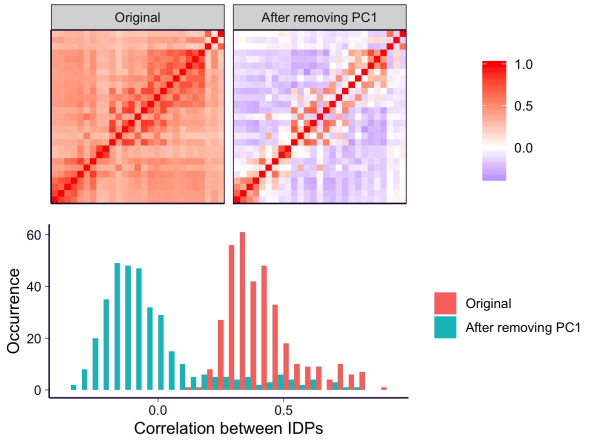

(d)

fig. S2. The correlation between IDPs for T1 modalities For each T1 modality, the correlation between brain IDPs are shown before and after removing PC1 by the heatmaps and the histrogram. Panel **a)** to **d)** show results for T1 modalities: gray matter volume of cortical regions, total volume of subcortical regions, gray matter volume of subcortical regions, and gray matter volume of cerebellum regions.

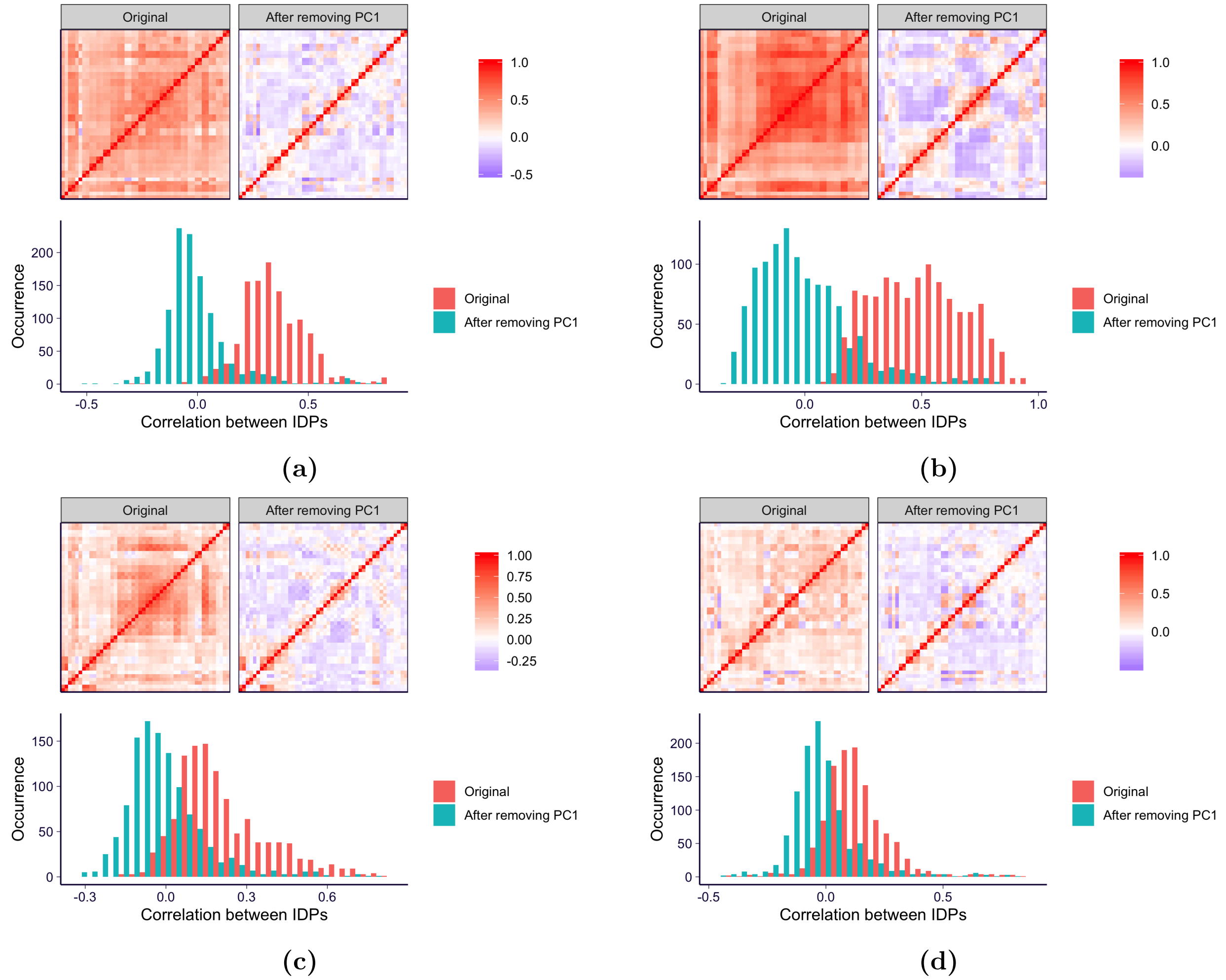

fig. S3. The correlation between IDPs for dMRI modalities For each dMRI modality, the correlation between brain IDPs are shown before and after removing PC1 by the heatmaps and the histrogram. Panel **a)** to **d)** show results for TBSS-based dMRI modalities: FA, ICVF, ISOVF, and OD.

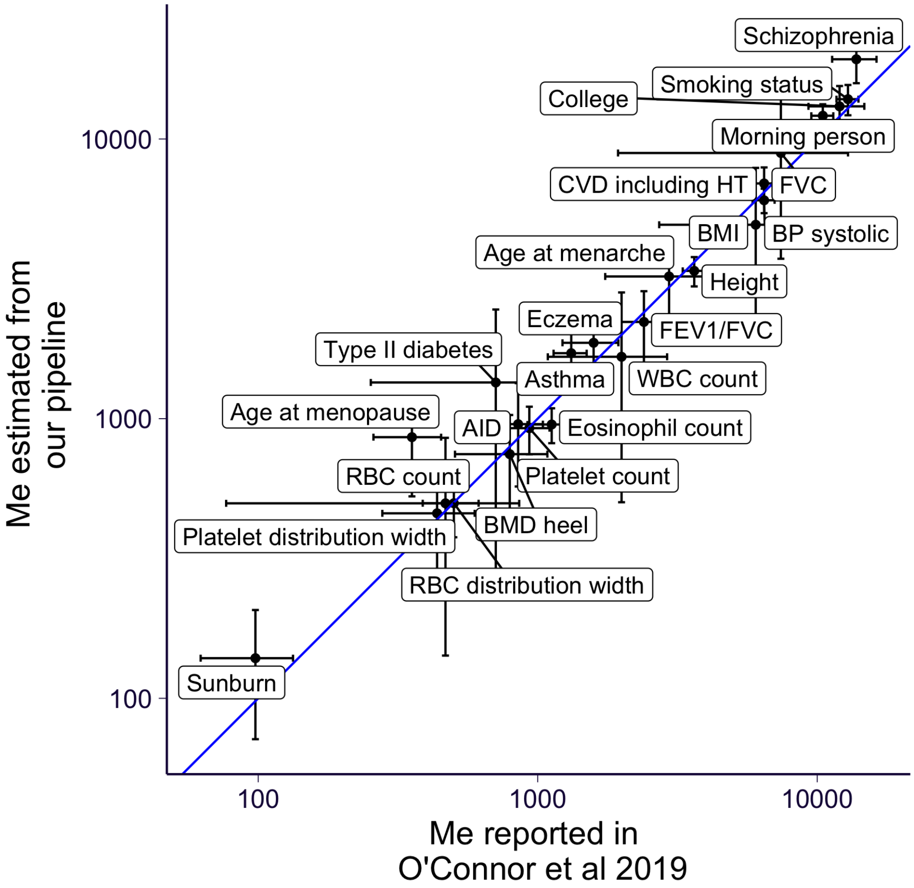

fig. S4. Comparing estimated M_e from [3] and our pipeline. Our M_e estimation pipeline is slightly different from the one being used in [3] (see more details in Methods). To check the robustness of our pipeline, we compared the estimated M_e from [3] (x-axis) and our pipeline (y-axis) for 24 traits. See definition of trait abbreviations from Table 1 and Table S4 of [3]. The error bar indicates the 95% confidence interval. The blue line is the identity line ($y = x$).

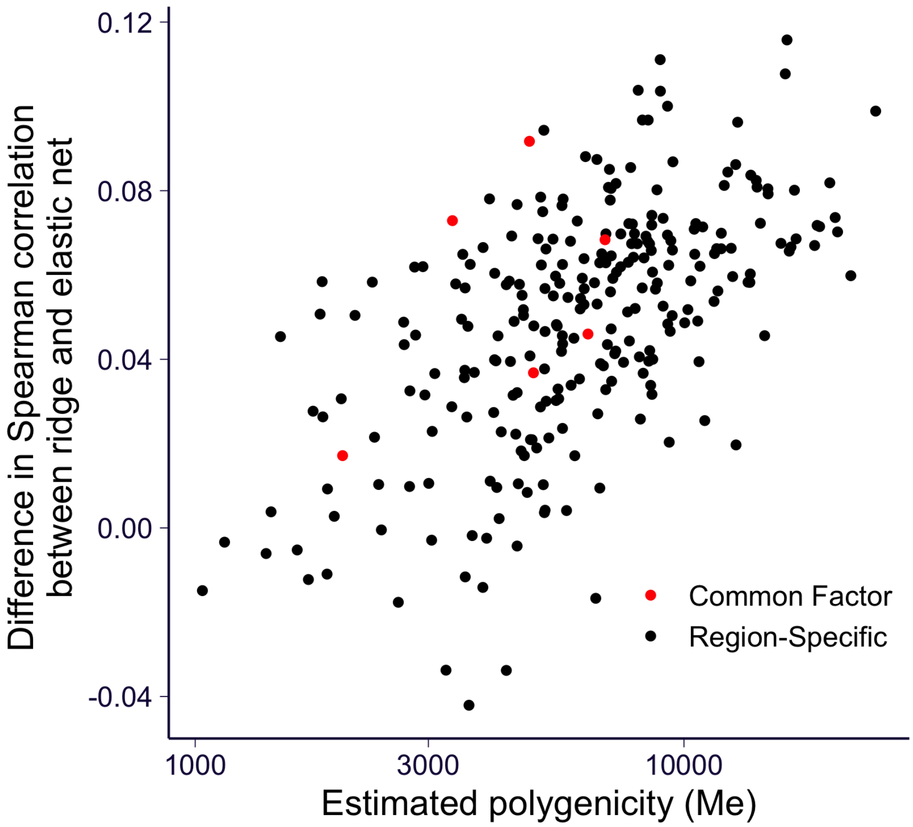

fig. S5. Increased performance of ridge predictors vs. polygenicity M_e. The x axis shows the estimated polygenicity, M_e, for the 522 IDPs with values significantly greater than 0 ($p < 0.05$). M_e is the "the effective number of independently associated SNPs", a proxy for number of causal SNPs. The y axis shows the difference in performance between ridge predictors and elastic net predictors (in terms of the difference in Spearman correlation). The IDP PCs are in red and the rest of the brain IDPs are in black.

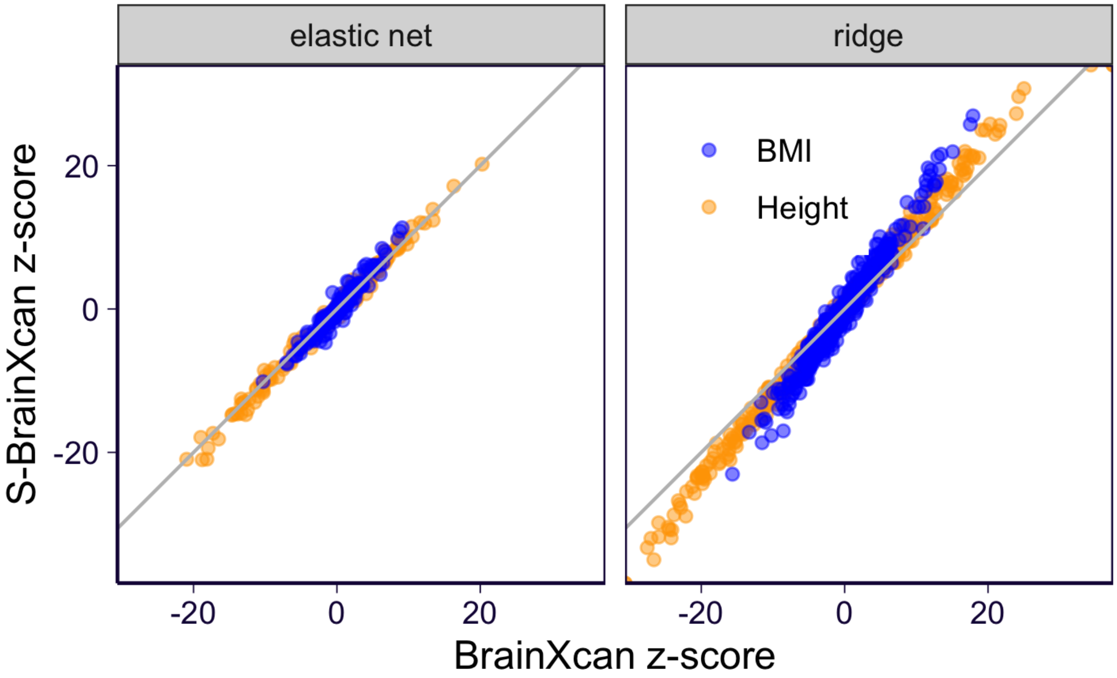

fig. S6. Comparing individual-level BrainXcan and S-BrainXcan results on UK Biobank standing height and BMI. We compare the BrainXcan z-scores of UK Biobank being calculated from the individual-level BrainXcan (on x-axis) and S-BrainXcan (on y-axis). For the ease of the comparison, the raw (S-)BrainXcan z-scores are shown (i.e. without adjustment). IDP models with prediction performance greater than 0.1 (Spearman correlation) are shown.

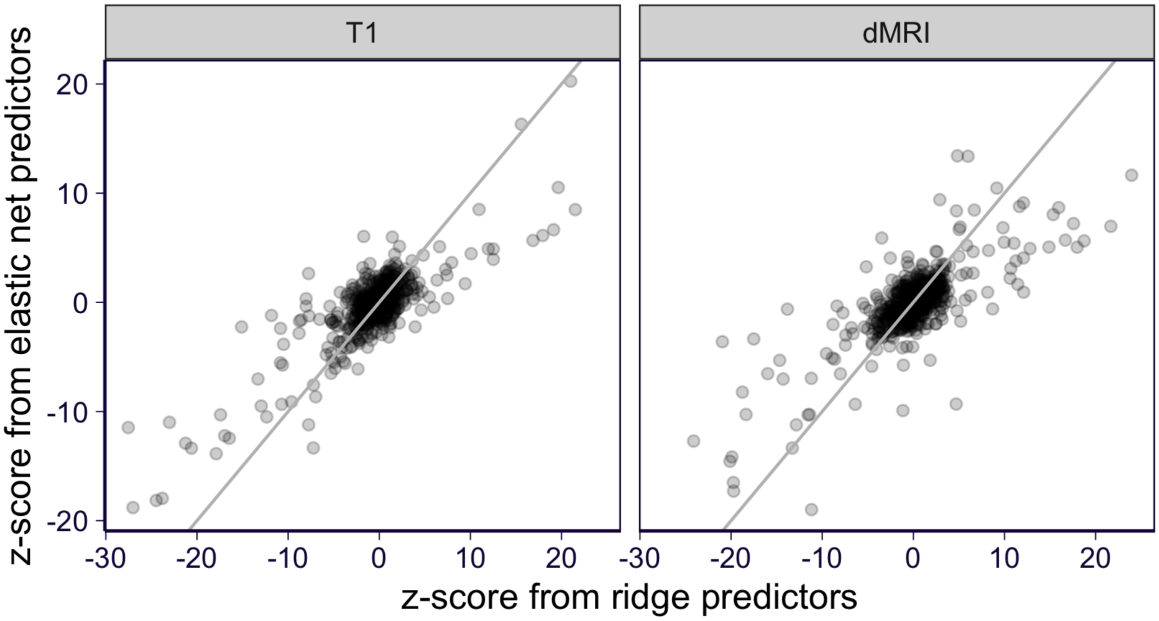

fig. S7. Comparing the ridge and elastic net based individual-level BrainXcan results. We compare the BrainXcan z-scores among the brain IDPs which have both ridge predictor and elastic net predictor with high quality (Spearman correlation > 0.1). The gray lines are the identity line ($y = x$). The Brainxcan z-score adjustment is only applicable to S-BrainXcan. So here, the raw BrainXcan z-scores are shown.

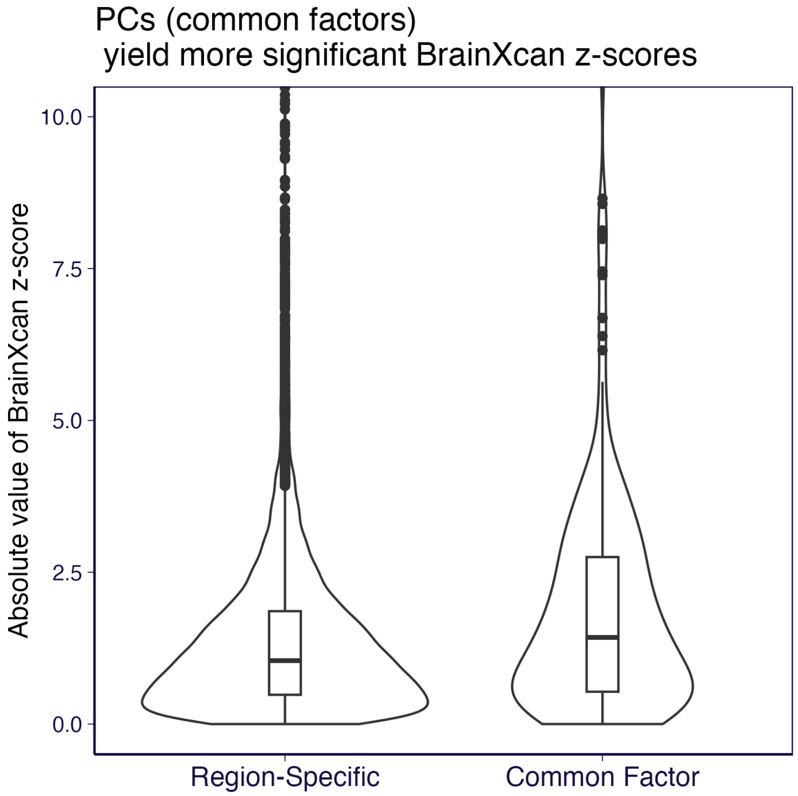

fig. S8. Comparing the S-BrainXcan significance between region-specific IDPs and common factors. S-BrainXcan significance is defined as the absolute value of BrainXcan z-score (with adjustment). We compare the S-BrainXcan significance (y-axis) of region-specific IDPs and common factors (PC1 of each IDP subtype) among the brain IDPs which have ridge predictor in high quality (Spearman correlation > 0.1).

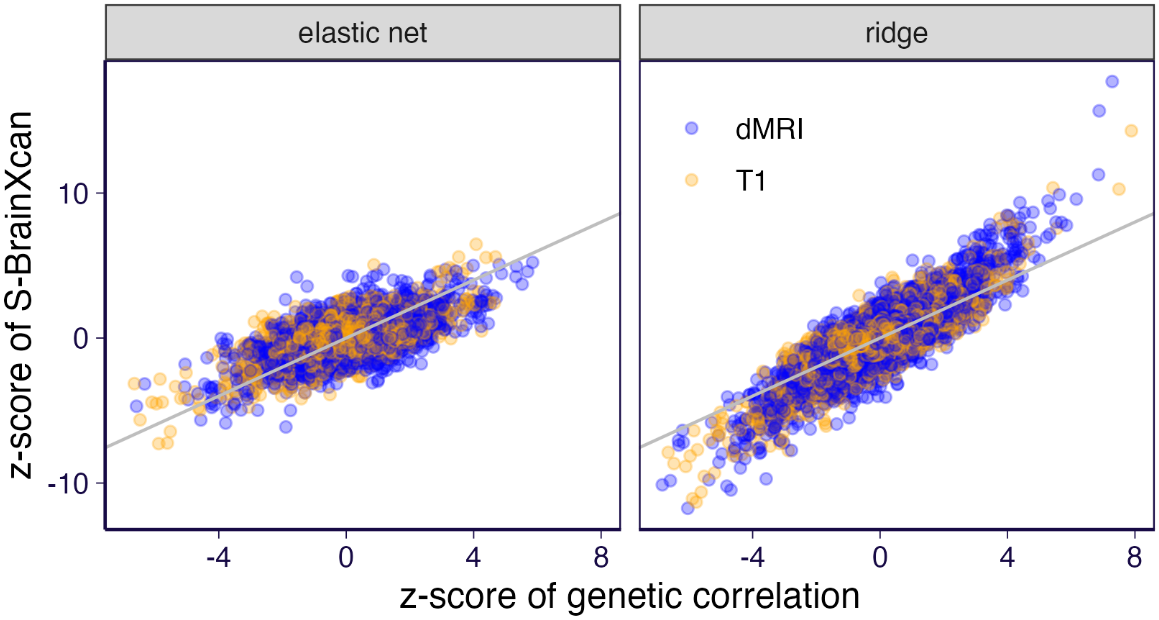

fig. S9. Comparing z-scores of the genetic correlation and S-BrainXcan. The z-scores of the genetic correlation (on x-axis) and the S-BrainXcan (with adjustment; on y-axis) are shown.

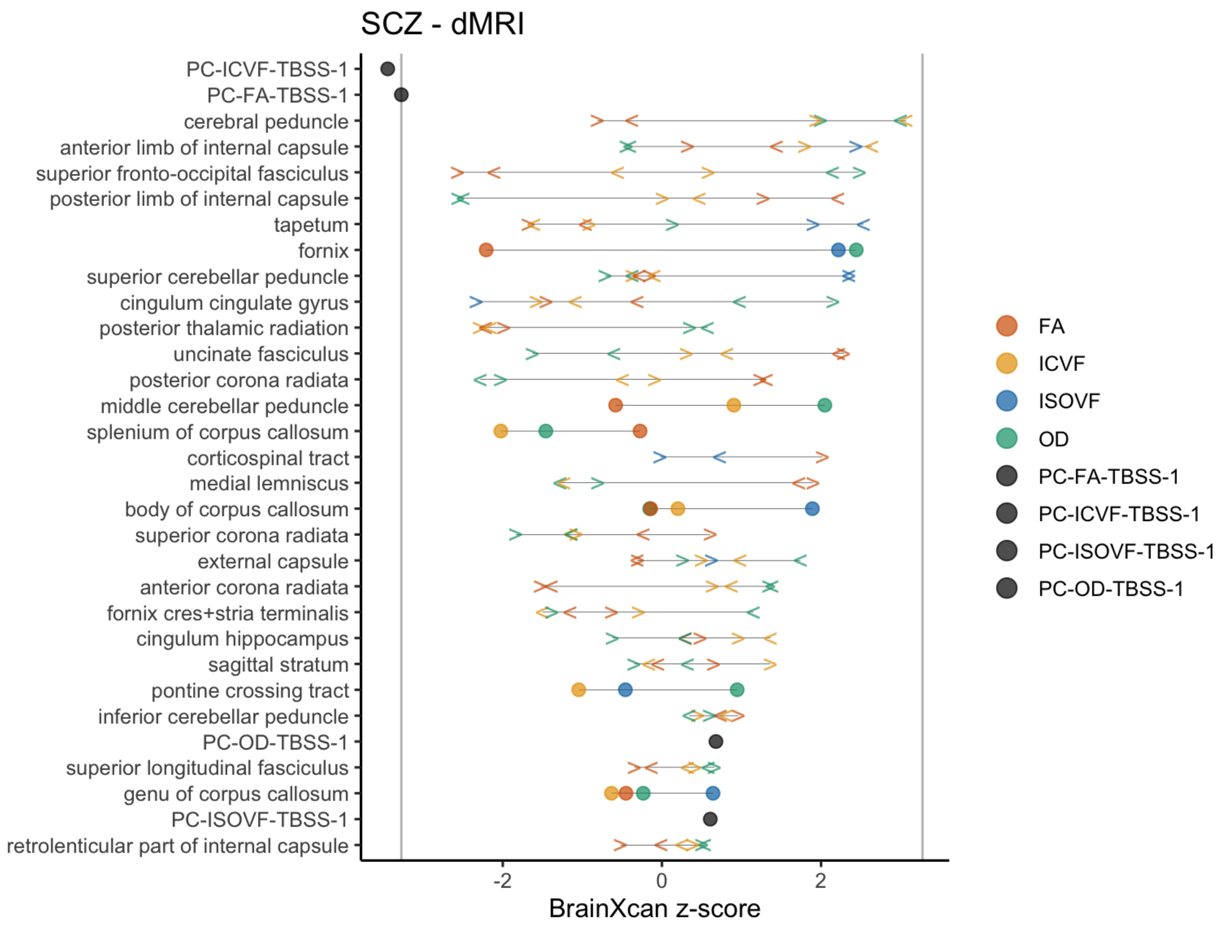

fig. S10. Schizophrenia risk association with diffusion MRI. Z-scores of region-specific feature associations with schizophrenia risk using GWAS effect sizes reported in [5]. Features starting with "PC" correspond to brain-wide properties (principal components of IDPs). FA: fractional anisotropy, ICVF: intracellular volume fraction, ISOVF: isotropic volume fraction, OD: orientation dispersion index. $<$ indicates left, $>$ indicates right, circles are used when sides are not defined. Vertical gray lines indicate significance threshold, FDR < 0.05.

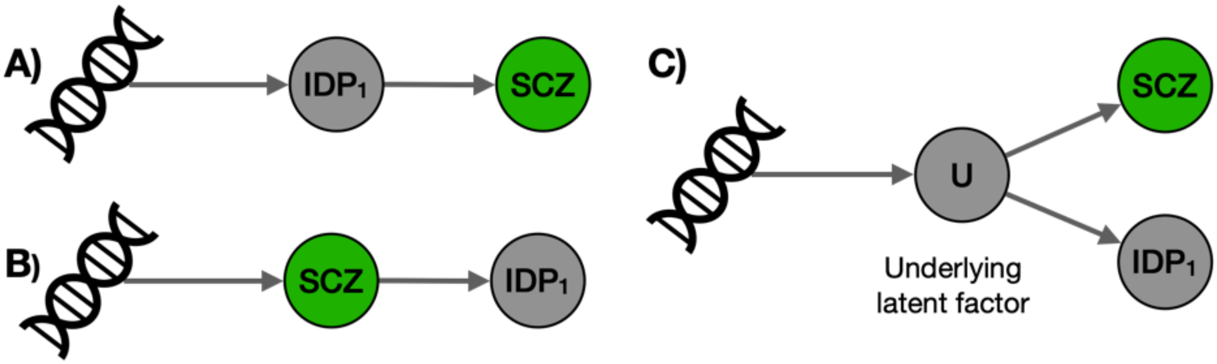

fig. S11. Mendelizan Randomization causal flow interpretation. Associations between brain features and the trait can arise from multiple mediating scenarios. We considered A) brain IDP alters trait, B) trait (disease status) alters brain IDP, C) underlying latent factor alters both trait and brain IDP. Given the power differential with current GWAS and reference image datasets, significant scenario B) may not rule out scenario A) or C). See discussion in text.

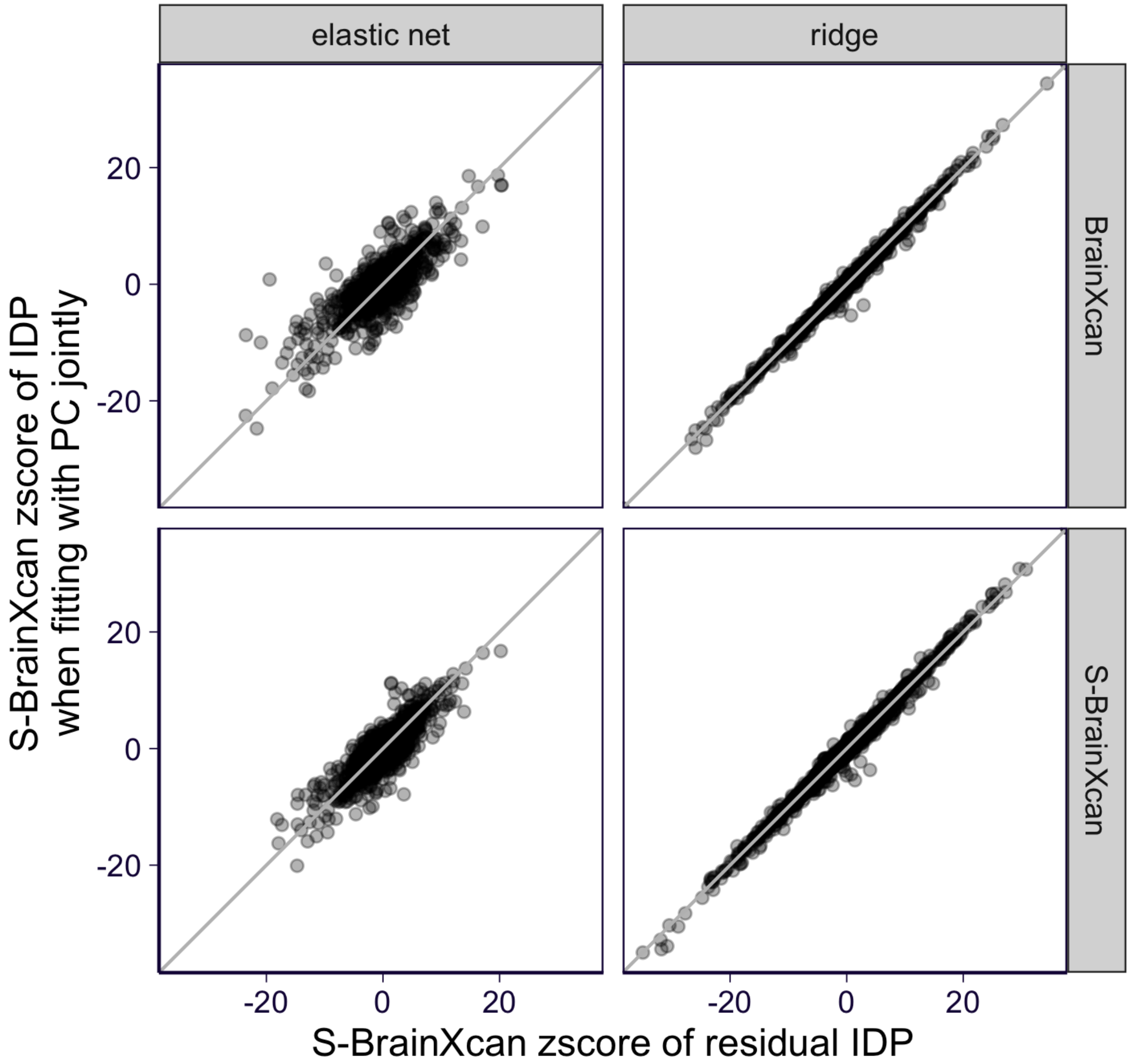

fig. S12. Comparing BrainXcan results from residual IDP and IDP adjusted by PC. We compare the BrainXcan z-scores obtained from $Y \sim$ resIDP (x-axis) and $Y \sim$ IDP + PC (y-axis). The top row shows results from individual-level BrainXcan and the bottom row shows results from summary BrainXcan. For the ease of the comparison, the raw (S-)BrainXcan z-scores are shown (i.e. without permutation-based adjustment). IDP models with prediction performance greater than 0.1 (Spearman correlation) are shown. The gray lines are the identity line ($y = x$). All GWASs are shown.

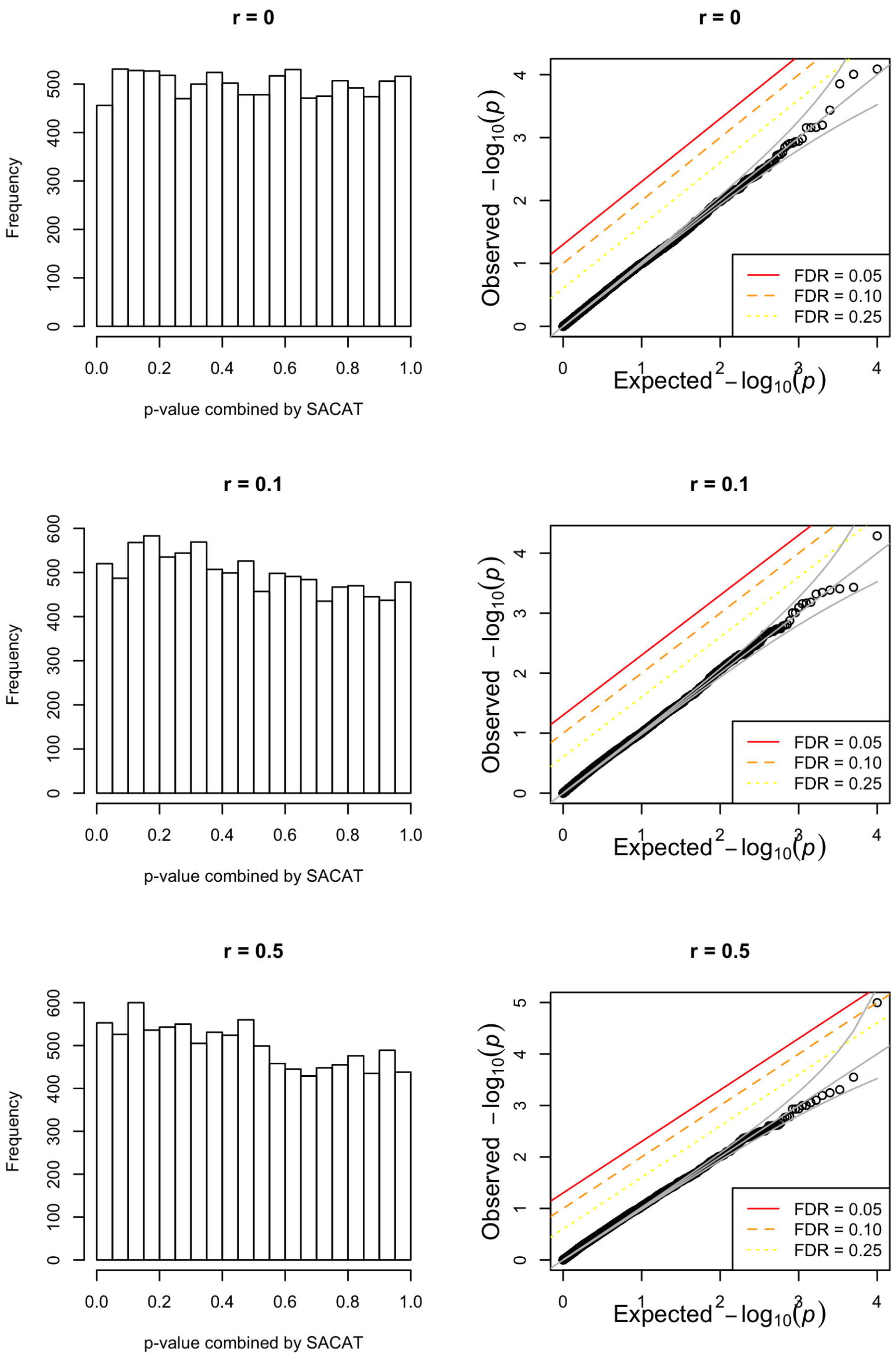

fig. S13. SACAT based p-value distribution under the global null. The simulation results of signed-ACAT (SACAT) are shown. The distribution of the SACAT-based meta-analyzed p-values are shown with histogram and QQ-plot (against uniform distribution). Each row shows the results from simulating the global null with dependency structure controlled by r. As r increases, the p-values being combined are more dependent and when $r = 0$, the p-values being combined are independent. These figures show that the SACAT is relatively well calibrated for different values of correlation between entries. See more simulation details in Supplementary Notes 3.

Supplementary Tables

variable	information	detail
age	54.9 (7.34)	range: 40–70
sex	52.5%/47.5%	raw count: 12814/11595

table S1. Demographic information of the IDP cohort. A summary of age and sex of the selected 24,409 individuals (IDP cohort) is shown. For age, **information** shows the average age with standard deviation of age in parentheses and **detail** shows the range of the age (min and max). For sex, **information** shows the fraction of each age among the cohort and **detail** shows the raw counts (females are listed first and followed by males)

table S2. UK Biobank brain IDPs being analyzed. This table contains the detailed information about the brain IDPs analyzed in this paper. The columns are listed below. **IDP** shows the IDP identifier used in this paper which uses the format of "IDP-XXXXX" with XXXXX being the Field ID in UK Biobank; **modality** indicates whether the IDP is from T1 or diffusion MRI; **subtype** lists the name of the IDP subtypes that is used in the paper to refer to different types of brain IDPs; **pc1_name** shows the name of the corresponding principal component of the subtype (this will be used as the IDP identifier for the IDP PC1); **region** shows the anatomical region of the MRI measurement; **side** indicates whether the measurement is from the left or right part of the brain; **measurement_type** shows the details of the measurement and the image processing procedure; **dmri_measure** indicates the type of statistic extracted from the dMRI measurement; **t1_anatomy_group** indicates the anatomical grouping for T1 MRI; **notes** shows the description of the IDP extracted from the UK Biobank database; **ukb_link** lists the link to the Data Field in UK Biobank website.

table S3. The heritability and poligenicity estimates of brain IDPs. IDP: IDP identifier; **h2** and **h2_SE**: The estimated heritability and the corresponding standard error; **Me** and **Me_SE**: The estimated polygenicity (defined as M_e in [3]) and the corresponding standard error.

table S4. The prediction performance of the ridge and elastic net predictors. It contains prediction performance (via cross-validation scheme) of the ridge regression and elastic net based genetic predictors of brain IDPs. **IDP**: IDP identifier; **model_name**: The type of prediction model being used (ridge regression or elastic net); **Spearman**: The Spearman correlation between the predicted and observed IDP values; **Pearson**: The Pearson correlation between the predicted and observed IDP values; **R2**: The R^2 measured from the predicted and observed IDP values; **is_kept**: An indicator showing whether the genetic predictor passes the quality control filter (e.g. Spearman correlation > 0.1) so that will be included in BrainXcan software.

table S5. The list of nine UK Biobank based phenotypes analyzed by individual-level BrainXcan. It contains the definition of the nine UK Biobank phenotypes used for individual-level BrainXcan. Some phenotypes were constructed from multiple UK Biobank fields and the aggregation method across these fields was either taking the sum (‘sum’) or taking logical OR (‘or’). For binary phenotype, we defined its value as 1 if the field takes the desired values indicated in **target_value**. Missing values were either treated as 0 or removed. **phenotype** shows the name of the defined phenotype; **ukb_field** shows UK Biobank fields from which the phenotype was constructed; **target_value**: For binary phenotypes, we defined its value as 1 if the field takes the desired value. For quantitative traits, we used the value as it is (labeled as ‘asis’); **aggregate_method**: For some phenotypes, they were constructed from multiple UK Biobank fields and the aggregation method across these fields was either taking the sum (‘sum’) or taking the logical OR (‘or’); **missing_values** shows how missing values in a field were handled (either treated as 0 or removed).

table S6. The list of 35 GWAS analyzed by S-BrainXcan. It contains the information about the 35 GWASs used in S-BrainXcan analysis. **phenotype**: The name of the phenotype; **phenotype_id**: The phenotype identifiers; **short_name**: The short names of the phenotypes; **sample_size**: The sample size of the GWAS. If there are different sample sizes for different SNPs, the mean sample size is shown; **portal**: The website from which the GWAS was downloaded; **filename**: The name of the raw GWAS file being downloaded.

table S7. S-BrainXcan results for 35 GWASs. model: The prediction model type of the IDP predictor (either ridge or elastic net); **IDP**: IDP identifier; **phenotype**: The name of the phenotype (defined in table S6); **bhat**: BrainXcan effect size estimate, $\hat{\beta}$; **pval**: BrainXcan p-value (without adjustment by permutation); **zscore**: BrainXcan z-score (without adjustment by permutation); **nsnp_used**: The number of variants used in S-BrainXcan computation; **nsnp_total**: The number of variants in the prediction model (**nsnp_used** should be no larger than **nsnp_total** but the closer the better since the difference is the number of variants being missed by the GWAS); **pval_corrected**: BrainXcan p-score after adjustment; **z_corrected**: The corresponding z-score computed from the adjusted BrainXcan p-value (**pval_corrected**).

table S8. LDSC based genetic correlation results for 35 GWASs. IDP: IDP identifier; **phenotype**: The name of the phenotype (defined in table S6); **rg**: Genetic correlation; **pval**: Genetic correlation p-value (null hypothesis: **rg** is zero); **zscore**: The corresponding z-score calculated from **pval**.

Supplementary Notes

1 Deriving bias of BrainXcan estimates

1.1 A generative model of IDP–phenotype association

Recall that we consider the following generative model describing the effect of brain IDPs within a subtype on a complex trait Y:

$$Y = \alpha \cdot L + \sum_k \beta_k \cdot F_k + \epsilon_Y, \ \epsilon_Y \sim N(0, \sigma^2) \tag{1}$$

where L represents a brain-wide factor that universally affects all IDPs with the subtype and F_k represents the IDP of a specific brain region within the subtype. Eq 1 assumes that both the brain-wide factor and regional IDPs may affect the complex trait and the effect sizes are α and β_k's respectively. In the BrainXcan analysis, we are interested in both brain-wide effects (α) and region-specific effects (β_k).

As described above, F_k is partially determined by the brain-wide factor and we introduce R_k to represent the rest of the variation in F_k which is region-specific. Furthermore, we assume that the region-specific variations R_k are independent to all other regions. More specifically, we assume

$$F_k = L + R_k, \ R_k \sim N(0, \sigma_k^2) \tag{2}$$

$$R_k \perp R_j, \ \forall k \neq j \tag{3}$$

In this setup, the relative contribution of L to F_k is determined by σ_k^2.

In practice, we don't observe F_k. Instead, MRI imaging pipeline measures a noisy version of F_k. We build genetic predictors using the noisy F_k so that we manage to (partially) capture the genetically determined variation in F_k. In other words, let IDP_k represent the predicted value of F_k and we assume that

$$\text{IDP}_k = F_k + \epsilon_k, \ \epsilon_k \sim N(0, \tau_k^2) \tag{4}$$

where τ_k^2 is the amount of noise when using IDP_k as the proxy for F_k.

To further simplify the derivation, we let $\tau_j^2 = t^2, \ \forall j$ and $\sigma_j^2 = s^2, \ \forall j$. These assumptions imply equal contribution of the brain-wide factor to all regions. And they also assume that the quality of F_k proxies (*i.e.* IDP_k) is the same across all regions. These assumptions simplify the notation and the qualitative conclusion still holds when this assumption is relaxed.

Notice that L is also an unobserved latent factor. We try to capture L by averaging over all IDP_k. In practice, we use the first principal component (PC1), which essentially is a weighted average of IDP_k, to approximate L. Using PC1 could account for the fact that the brain-wide factor does not contribute equally to all regions and predictor quality is not the same across all regions. But since we assume equal contribution for L and equal quality for IDP_k, we simply use unweighted average of IDP_k as the proxy for L, which is shown below:

$$\text{PC} = \frac{1}{m} \sum_k \text{IDP}_k \tag{5}$$

$$= L + \frac{1}{m}\sum_k (R_k + \epsilon_k) \tag{6}$$

where m is the number of regions within the subtype being considered.

1.2 Variances and covariances among variables

Here we list the variance and covariance among model variables:

$$\text{Cov}(F_i, F_j) = \begin{cases} 1 & , i \neq j \\ 1 + s^2 & , i = j \end{cases}$$

$$\text{Cov}(\text{IDP}_i, \text{IDP}_j) = \begin{cases} 1 & , i \neq j \\ 1 + s^2 + t^2 & , i = j \end{cases} \tag{7}$$

$$\text{Var}(\text{PC}) = 1 + \frac{1}{m^2}\sum_k (s^2 + t^2) \tag{8}$$

$$\text{Cov}(\text{PC}, \text{IDP}_j) = 1 + \frac{1}{m}(s^2 + t^2) \tag{9}$$

$$\text{Cov}(L, \text{IDP}_j) = \text{Cov}(L, F_j) = 1$$

$$\text{Cov}(Y, \text{IDP}_j) = \alpha + \sum_k \beta_k + s^2 \beta_j \tag{10}$$

$$\text{Cov}(Y, \text{PC}) = \alpha + \sum_k \beta_k + \frac{1}{m}\sum s^2 \beta_k \tag{11}$$

1.3 Biases of the BrainXcan associations

As described in Supplementary Notes 1.1, IDP_k is a proxy of F_k and PC is a proxy of L. In BrainXcan analysis, we fit linear regression model $Y \sim \text{IDP}_k + \text{PC}$, in which we seek to test whether there exists the region-specific effect ($\beta_k \neq 0$) . Thus, we focus on testing if the coefficient of IDP_k is zero. Similarly, we also fit $Y \sim \text{PC}$ to test for a brain-wide effect of the subtype. In this section, we derive the expected value of these coefficients (coefficient of IDP_k in $Y \sim \text{IDP}_k + \text{PC}$ and coefficient of PC in $Y \sim \text{PC}$) to determine how they related to the parameters of interest (β_k and α).

Coefficient of IDP_k

Consider fitting the linear model $Y \sim \text{IDP}_k + \text{PC}$. The coefficient of IDP_k is

$$\begin{bmatrix} \text{coef IDP}_k \\ \text{coef PC} \end{bmatrix} = \begin{bmatrix} \widehat{\text{Var}}(\text{IDP}_k) & \widehat{\text{Cov}}(\text{IDP}_k, \text{PC}) \\ \widehat{\text{Cov}}(\text{IDP}_k, \text{PC}) & \widehat{\text{Var}}(\text{PC}) \end{bmatrix}^{-1} \begin{bmatrix} \widehat{\text{Cov}}(\text{IDP}_k, Y) \\ \widehat{\text{Cov}}(\text{PC}, Y) \end{bmatrix} \tag{12}$$

Taking the expected value of the coefficient, we have

$$\text{E}\left(\begin{bmatrix} \text{coef IDP}_k \\ \text{coef PC} \end{bmatrix}\right) = \begin{bmatrix} \text{Var}(\text{IDP}_k) & \text{Cov}(\text{IDP}_k, \text{PC}) \\ \text{Cov}(\text{IDP}_k, \text{PC}) & \text{Var}(\text{PC}) \end{bmatrix}^{-1} \begin{bmatrix} \text{Cov}(\text{IDP}_k, Y) \\ \text{Cov}(\text{PC}, Y) \end{bmatrix} + O_p\left(\frac{1}{n}\right) \tag{13}$$

, where n is the sample size of the linear regression. And the $O_p(\cdot)$ is introduced when plugging-in variances and covariances in the places of sample variances and covariances (see more detailed discussion in [1] Appendix A).

Substituting Eq 7-11 for quantities in Eq 13, we have

$$\mathrm{E}(\text{coef IDP}_k) = \underbrace{\frac{s^2}{s^2+t^2}}_{\text{Attenuation bias}} \cdot \left[\beta_k - \underbrace{\frac{1}{m-1}\sum_{j\neq k}\beta_j}_{\text{Collider effect}}\right] + O\left(\frac{1}{n}\right) \tag{14}$$

$$= \beta_k - \frac{t^2}{s^2+t^2}\cdot\beta_k - \frac{s^2}{s^2+t^2}\sum_{j\neq k}\frac{\beta_j}{m-1} + O\left(\frac{1}{n}\right) \tag{15}$$

We note that there are two sources of bias. First, $\frac{s^2}{s^2+t^2}$ term is introduced by the fact that IDP_k is a noisy version of the actual affecting variable F_k, which is the so called attenuation bias. Second, $\frac{1}{m-1}\sum_{j\neq k}\beta_j$ term is introduced by the fact that PC is used instead of L as a covariate. As shown in Eq 6, PC captures not only L but R_k's which makes PC a collider variable in testing association between Y and IDP_k.

In summary, the coefficient of IDP_k in $Y \sim \text{IDP}_k + \text{PC}$ mainly captures the effect of region k on Y (β_k), but it also captures the average effect from all other regions. In a situation where only a few regions have non-zero effects, the second term is usually small.

Coefficient of PC

Consider fitting the linear model $Y \sim \text{PC}$. The coefficient of PC is

$$\text{coef PC} = \frac{\widehat{\text{Cov}}(\text{PC}, Y)}{\widehat{\text{Var}}(\text{PC})} \tag{16}$$

Similarly to Eq 13, to work out the expected value of PC coefficient, we plug-in variances and covariances in the places of sample variances and covariances.

$$\mathrm{E}(\text{coef PC}) = \frac{\text{Cov}(\text{PC}, Y)}{\text{Var}(\text{PC})} + O(\frac{1}{n}) \tag{17}$$

$$= \frac{m\alpha + (s^2+m)\sum_j \beta_j}{s^2+t^2+m} \tag{18}$$

$$= \alpha + \sum_j \beta_j + O(\frac{1}{m}) + O(\frac{1}{n}) \tag{19}$$

In summary, the coefficient of PC in $Y \sim \text{PC}$ captures the overall effect of the subtype ($\alpha + \sum_j \beta_j$).

2 Using IDP residual instead of fitting IDP and PC jointly

Fitting IDP_k and PC jointly requires estimating the sample covariance between the predicted IDP_k and PC. In summary-based BrainXcan, this estimation relies on an external LD panel, which may

contain some noise or even error, especially when the LD panel is not representative of the GWAS cohort. In this case, the joint model fitting is sensitive to the quality of the sample covariance estimation. To ensure the robustness of the BrainXcan test, we take an alternative approach which avoid estimating the sample covariance.

In the alternative approach, we fit $Y \sim \text{resIDP}_k$ instead, where resIDP_k is the predicted value of IDP_k residual (after regressing out PC). In this section, we show that the coefficient of IDP_k in the joint model $Y \sim \text{IDP}_k + \text{PC}$ is approximately equivalent to the result of a two-step approach:

1. Regress out PC from IDP_k and keep the residual r_k.
2. Obtain coefficient of r_k in $Y \sim r_k$.

First of all, we can calculate r_k from model $\text{IDP}_k \sim \text{PC}$.

$$r_k = \text{IDP}_k - a_k \cdot \text{PC} \tag{20}$$

$$a_k = \frac{\widehat{\text{Cov}}(\text{IDP}_k, \text{PC})}{\widehat{\text{Var}}(\text{PC})} \tag{21}$$

So, we have

$$\text{Cov}(Y, r_k) = \text{Cov}(Y, \text{IDP}_k) - a_k \cdot \text{Cov}(Y, \text{PC}) \tag{22}$$

$$\text{Var}(r_k) = \text{Var}(\text{IDP}_k) - 2 \cdot a_k \cdot \text{Cov}(\text{IDP}_k, \text{PC}) + a_k^2 \cdot \text{Var}(\text{PC}) \tag{23}$$

And in step 2, we can obtain the coefficient of r_k from $Y \sim r_k$.

$$\text{coef } r_k = \frac{\widehat{\text{Cov}}(Y, r_k)}{\widehat{\text{Var}}(r_k)} \tag{24}$$

$$\text{E}(\text{coef } r_k) \approx \frac{\text{Cov}(Y, r_k)}{\text{Var}(r_k)} \tag{25}$$

$$= \frac{s^2}{s^2 + t^2} \cdot \left[\beta_k - \frac{1}{m-1} \sum_{j \neq k} \beta_j\right] \tag{26}$$

Comparing Eq 14 and 26, we can conclude that $\text{E}(\text{coef IDP}_k) \approx \text{E}(\text{coef } r_k)$. And this result indicates that regressing the outcome Y on IDP_k and PC jointly is approximately equivalent to regressing Y on the residual of IDP_k.

In practice, instead of calculating the residual IDP_k by regressing out predicted PC from predicted IDP_k (which still relies on covariance between PC and IDP_k), we build the genetic predictor of IDP_k residual and predict the residual IDP_k (which is called resIDP_k) directly. We show empirically that the coefficient from $Y \sim \text{resIDP}_k$ is similar to coef IDP_k (fig. S12).

3 Aggregating Mendelian Randomization test results by extending the Aggregated Cauchy Association test (ACAT) method

The ACAT method [2] is a "meta-analysis" approach that leverages the fact that averages of possible correlated Cauchy random variables are Cauchy-distributed [4]. The method ignores the

direction of the association since it uses p-values alone. However, it is obvious that two studies with opposite direction of effects should somewhat cancel each other and yield a less significant p-value when combined. Here, we propose an approach to take the sign into account.

ACAT combines potentially dependent p-values as follows:

$$T = \sum_i \tan[(\frac{1}{2} - p_i) \cdot \pi] \tag{27}$$

$$p_{\text{ACAT}} = \frac{1}{2} - \frac{\arctan(T/N)}{\pi} \tag{28}$$

where N is the total number of p-values being combined and T is the test statistic, which follows Cauchy distribution under the null. So, we want to apply ACAT to combine the results of multiple Mendelian randomization tests.

In the original use case of the ACAT method, p-values are combined without considering the direction of the effect. In our specific example, various Mendelian randomization tests yield p-values (p_i) and direction of the effects (s_i). Therefore, we want to construct a variation of ACAT such that the direction of the effect is considered. Specifically, to combine p-values $p_1, \cdots, p_N$ with signs $s_1, \cdots, s_N$, if we assume the "+1" direction is the direction of true signal, we take the signs into consideration by modifying Eq 27:

$$T_{+1} = \sum_i \{s_i \cdot \tan[(\frac{1}{2} - p_i) \cdot \pi]\}, \tag{29}$$

or for the negative direction

$$T_{\text{-}1} = -\sum_i \{s_i \cdot \tan[(\frac{1}{2} - p_i) \cdot \pi]\}. \tag{30}$$

More generally, considering $s \in \{-1, +1\}$ as the true direction:

$$T_s = s \cdot \sum_i \{s_i \cdot \tan[(\frac{1}{2} - p_i) \cdot \pi]\}. \tag{31}$$

In practice, because both "+1" and "-1" directions are possible, we should test both and combine the two p-values at the end. For this purpose, we propose the following meta-analysis approach, signed ACAT (SACAT), which takes p-values $p_1, \cdots, p_N$ with signs $s_1, \cdots, s_N$ and return the combined p-value and sign:

$$p_s = \frac{1}{2} - \frac{\arctan(T_s/N)}{\pi} \tag{32}$$

$$p_{\text{SACAT}} = 2 \cdot \min_s p_s \tag{33}$$

$$s_{\text{SACAT}} = \arg\min_s p_s \tag{34}$$

The p_{SACAT} is derived using the fact that $T_{+1} = -T_{\text{-}1}$ implies $p_+ + p_- = 1$ and therefore $P\{\min(p_+, p_-) < u\} = P\{\min(p_+, (1 - p_+)) < u\} = P\{\min(p_-, (1 - p_-)) < u\} = 2u$ with u taking values between 0 and 1/2.

To examine the calibration of SACAT, we perform a simulation study in which we simulate from the global null using the procedure in below:

1. For $i = 1, \cdots, 20$, we simulate $Z_i \sim N(0,1)$ where $\text{Cov}(Z_i, Z_j) = r$ except $\text{Cov}(Z_1, Z_j) = -r$.
2. Calculate $p_i = 2 \times \Pr(|Z| > Z_i; Z \sim N(0,1))$.
3. Repeat the above two steps 10000 times each for $r = 0, 0.1, 0.5$.

The dependence among p-values is induced by $r \neq 0$. Fig. S13 shows that the proposed SACAT is well calibrated under the global null since the resulting meta-analyzed p-values are roughly uniformly distributed and the points in the qq-plot against the null distribution lie on the identity line for all three values of dependence r.

4 Caveats on interpreting Mendelian randomization results

There are two caveats that need to be considered when interpreting Mendelian randomization results. One is that we first select the IDP–trait pair based on their association. Therefore, the p-values of the Mendelian randomization will not be well-calibrated, i.e., even under the null of no causal link, the p-value will be biased towards smaller values. Given this bias, we should not use the Mendelian randomization p-values to claim significance, but as scores to discern between possible direction of the causal flow.

The second caveat relates to the power difference between reference image and GWAS studies. Currently, reference image data have much smaller sample sizes (n $\sim$ 30K) compared to GWAS studies of complex traits (n $\sim$ 100K to 1M). We consider three main mediating scenarios depicted in fig. S11. In scenario A, the brain feature mediates the genetic association with the phenotype, i.e., genetic risk factors alter the brain feature which in turn alters the risk for the phenotype. In scenario B, genetic factors affect the phenotype which in turn alter the brain feature. In scenario C, genetic factors affect an underlying latent factor which alter both the phenotype and the brain feature.

A significant result in scenario A and not in scenario B can be interpreted as evidence that the brain feature alteration is affecting the phenotype given the higher power of GWAS studies in general. However, a significant result in scenario B and non-significant result in scenario A could simply mean that the instruments (strongly associated SNPs and their effect sizes) for the brain feature are not reliable enough to yield significance. In this case, scenario B should be considered supported by the data, but scenarios A and C should not be ruled out.

References

[1] Hugues Aschard, Bjarni J Vilhjálmsson, Amit D Joshi, Alkes L Price, and Peter Kraft. Adjusting for heritable covariates can bias effect estimates in genome-wide association studies. *The American Journal of Human Genetics*, 96(2):329–339, 2015.

[2] Yaowu Liu, Sixing Chen, Zilin Li, Alanna C Morrison, Eric Boerwinkle, and Xihong Lin. Acat: A fast and powerful p value combination method for rare-variant analysis in sequencing studies. *The American Journal of Human Genetics*, 104(3):410–421, 2019.

[3] Luke J O'Connor, Armin P Schoech, Farhad Hormozdiari, Steven Gazal, Nick Patterson, and Alkes L Price. Extreme polygenicity of complex traits is explained by negative selection. *The American Journal of Human Genetics*, 105(3):456–476, 2019.

[4] Natesh S Pillai and Xiao-Li Meng. An unexpected encounter with cauchy and lévy. *aos*, 44(5):2089–2097, October 2016.

[5] Stephan Ripke, James TR Walters, Michael C O'Donovan, Schizophrenia Working Group of the Psychiatric Genomics Consortium, et al. Mapping genomic loci prioritises genes and implicates synaptic biology in schizophrenia. *MedRxiv*, 2020.
